# Structure–Activity Relationship of *N*-Ethyl-Hexedrone Analogues: Role of the α-Carbon Side-Chain Length in the Mechanism of Action, Cytotoxicity, and Behavioral Effects in Mice

**DOI:** 10.1021/acschemneuro.2c00772

**Published:** 2023-02-03

**Authors:** Núria Nadal-Gratacós, Edwin Ríos-Rodríguez, David Pubill, Xavier Batllori, Jorge Camarasa, Elena Escubedo, Xavier Berzosa, Raúl López-Arnau

**Affiliations:** † Pharmaceutical Chemistry Group (GQF), IQS School of Engineering, Universitat Ramon Llull, 08017 Barcelona, Spain; ‡ Department of Pharmacology, Toxicology and Therapeutic Chemistry, Pharmacology Section and Institute of Biomedicine (IBUB), Faculty of Pharmacy, University of Barcelona, 08028 Barcelona, Spain

**Keywords:** synthetic cathinones, new psychoactive substances, reward, psychostimulant, cytotoxicity, anxiety

## Abstract

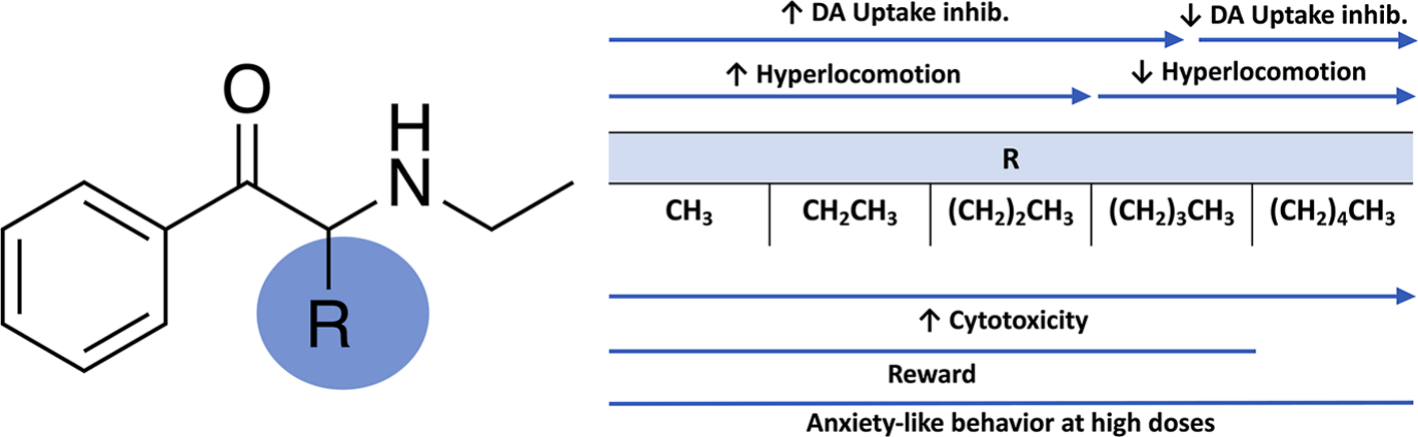

Synthetic cathinones are β-keto amphetamine derivatives whose appearance has increased dramatically in the past decades. *N*-Ethyl substituted cathinones have been proven to potently inhibit dopamine (DA) uptake and induce psychostimulant and rewarding effects in mice. However, little is known about the influence of the alpha-carbon side-chain length of *N*-ethyl cathinones on their pharmacological and toxicological effects. Thus, the aim of this study was to synthesize and investigate the in vitro and in vivo effects of five *N*-ethyl substituted cathinones: *N*-ethyl-cathinone (NEC), *N*-ethyl-buphedrone (NEB), *N*-ethyl-pentedrone, *N*-ethyl-hexedrone (NEH), and *N*-ethyl-heptedrone. HEK293 cells expressing the human DA or serotonin transporter (hDAT and hSERT) were used for uptake inhibition and binding assays. PC12 cells were used for the cytotoxicity assays. Swiss CD-1 mice were used to study the in vivo psychostimulant, anxiogenic, and rewarding properties. Our results show that all tested cathinones are able to inhibit DA uptake and are DAT-selective. The potency of DA uptake inhibitors increases with the elongation of the aliphatic side chain from methyl to propyl and decreases when increasing from butyl to pentyl, which correlates with an inverted *U*-shape psychostimulant response in mice at the medium dose tested. On the other hand, an increase in the α-carbon side-chain length correlates with an increase in the cytotoxic properties in PC12 cells, probably due to better membrane penetration. Moreover, all the cathinones tested have shown higher cytotoxicity than methamphetamine. Finally, our study not only demonstrated the rewarding properties of NEC and NEB but also the anxiety-like behavior induced at high doses by all the cathinones tested.

## 1 Introduction

**Figure 1:**
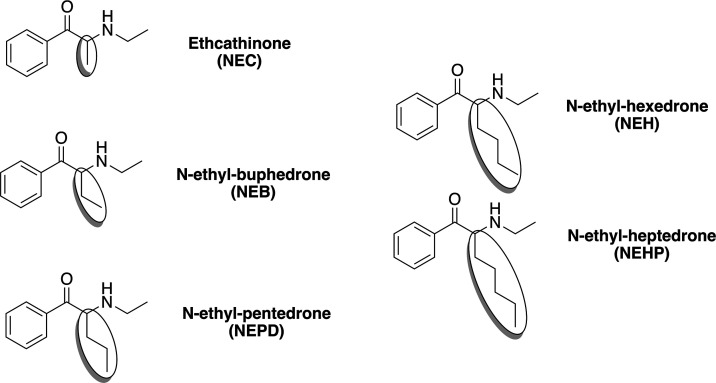
Chemical structure of NEC, NEB, NEPD, NEH, and NEHP.

The popularity of synthetic cathinones, a subclass of new psychoactive substances (NPS), has importantly increased during the past decades as they are often mistakenly seen as legal, safer, and even less addictive alternatives to other classic psychostimulants such as cocaine, amphetamine, or 3,4-methylenedioxymethamphetamine.^1−5^ Moreover, novel synthetic cathinones are constantly emerging by performing structural modifications to the chemical structure of cathinone, leading to an enormous group of compounds whose pharmacological and toxicological effects are unknown by users, researchers, and clinicians. In this sense, structure–activity relationship studies performed by our research group and others have demonstrated the potency of *N*-ethyl substituted cathinones inhibiting dopamine (DA) uptake as well as inducing psychostimulant and rewarding effects,^6−10^ pointing to a public health concern. Thus, the present study is focused on five different *N*-ethyl substituted cathinones, *N*-ethyl-cathinone (NEC) (ethcathinone), *N*-ethyl-buphedrone (NEB), *N*-ethyl-pentedrone (NEPD), *N*-ethyl-hexedrone (NEH), and *N*-ethyl-heptedrone (NEHP) (Figure 1), which only differ in the alpha-carbon side-chain length, a common structural modification found in the NPS’s market. Furthermore, these substances have recently been reported and identified in the illicit drug market,^11−17^ and intoxications and even fatalities have been associated with their use and abuse.^14,18−20^ In fact, NEH has been classified as Schedule II controlled substance under the United Nations Convention on Psychotropic Substances^21,22^ due to its prevalence and dramatic increase in the number of identifications and reports in the past years.^11,17^

Similar to other synthetic cathinones, ethcathinone (NEC), NEPD, and NEH are potent DA uptake inhibitors and are able to stimulate locomotor activity in rodents.^6−8,23−26^ Studies suggest that NEH also substitutes for the discriminative stimulus effects of methamphetamine and cocaine.^23^ Unfortunately, information about the pharmacological and toxicological properties of these compounds is still limited, especially for NEB and NEHP.

Regarding structure–activity relationship studies, the length of the α-carbon chain of α-pyrrolidinophenones, which are synthetic cathinones with a five-member nitrogen-containing ring on the α-carbon, is correlated with DA uptake inhibition potency, showing the compounds containing tails with three to six carbons very high affinity for the DA transporter (DAT).^27−29^ Similar to the pyrrolidino series, the length of the α-carbon chain affected the potency of pentylone analogues containing the methylenedioxy moiety.^7,30^ Moreover, the α-alkyl-side-chain length of pentylone analogues is inversely associated with transporter-gating efficacy at the serotonin (5-HT) transporter (SERT).^30^

It is well established that synthetic cathinones possess reduced neurotoxic potential when compared to their amphetamine counterparts,^31−33^ but cathinone’s toxicity must not be underestimated. In fact, other reports also point to the potential neurotoxic effects of synthetic cathinones in humans.^34−38^ Some in vitro studies suggest that the increased α-carbon length chain might be a key point for the cytotoxicity profile of α-pyrrolidinophenones.^39,40^ Moreover, the same study also demonstrated that the substitution of the pyrrolidine ring by secondary amine analogues resulted in increased cytotoxic activity.^40^ However, no studies focused on the neuropharmacological and toxicological properties of synthetic cathinones with a wide spectrum of α-alkyl-side-chain length and ethylamino-substitution have been reported yet.

Therefore, with the endeavor to identify the role of the aliphatic side-chain length and study the neuropharmacological and toxicological profile of ethylamino-substituted cathinones, the aim of the present work was to (i) synthesize the previously mentioned synthetic cathinones: NEC, NEB, NEPD, NEH, and NEHP, (ii) study their interaction with DAT and SERT, (iii) determine the cytotoxic potential in PC12 cells, and (iv) study the psychostimulant, anxiety-like effects and rewarding properties of these cathinones in mice.

## 2 Results and Discussion

### 2.1 Monoamine Uptake Inhibition and Transporter Affinity Studies

**Figure 2:**
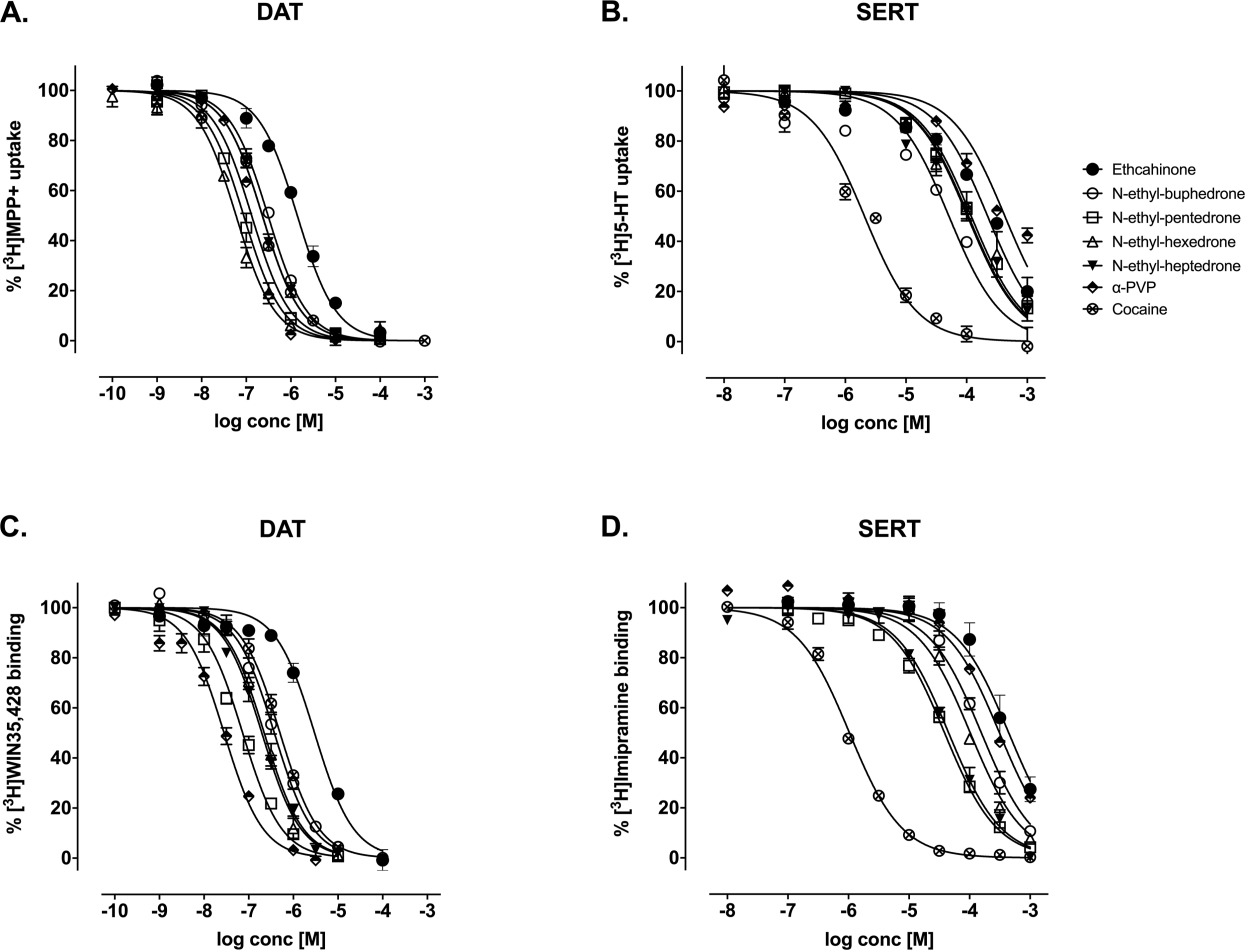
Competition binding curves of NEC, NEB, NEPD, NEH, NEHP, α-PVP, and cocaine on [^3^H]MPP^+^ uptake at DAT and [^3^H]5-HT uptake at SERT (panel A,B) and [^3^H]WIN35,428 binding at DAT and [^3^H]Imipramine binding at SERT (panel C,D) in transfected HEK293 cells. Data are expressed as a percentage of control uptake (mean ± SEM) of four independent experiments carried out in triplicate.

Due to the ability of *N*-ethyl-substituted cathinones^8^ to inhibit monoamine uptake and produce psychostimulant and rewarding effects, the study of the influence of the length of the α-carbon chain in such class of compounds seems necessary to better define their pharmacological and toxicological effects. In the present study, we first examined the ability of the five selected *N*-ethyl-substituted cathinones (see Figure 1) to act at the monoamine transporters DAT and SERT. Concentration–response curves are presented in Figure 2 (panel A–B) and the corresponding IC_50_ values and hDAT/hSERT inhibition ratios are summarized in Table 1. Our results show that all the cathinones tested to act as potent DAT inhibitors. Of particular interest, and in accordance with others’ results,^24,28^ the potency at inhibiting DA uptake was higher when increasing the length of the aliphatic side chain from methyl to propyl. On the other hand, NEPD and NEH presented similar inhibition potency, but the addition of an extra carbon (NEHP) resulted in a decreased potency for DA uptake inhibition but still higher in comparison with NEC. Moreover, all the compounds tested showed similar potency inhibiting DA uptake to α-PVP, but not NEC, which showed approximately a 10-fold reduction. In addition, all cathinones presented none or little activity in inhibiting SERT (see Table 1), altogether resulting in high DAT/SERT inhibition ratios, which have been associated with abuse liability.^30,41−43^ All the cathinones under study have shown higher DAT/SERT ratios than cocaine but lower than α-PVP, with NEPD and NEH being the most DA-selective ones of the compounds tested, suggesting a high risk of abuse potential. Moreover, Gannon and co-workers^44^ demonstrated that a longer α-alkyl side chain positively correlated with the reinforcing potency of α-PVP and MDPV derivatives (from methyl to propyl),^44^ which at the same time correlated with higher potency at inhibiting DAT.^27^ In our study, DAT/SERT ratio increases with the elongation of the α-carbon side chain from methyl to butyl, which agrees with this finding, but starts decreasing in the presence of an extra carbon (Table 1).

**Table 1:** Monoamine Uptake Inhibition and Transporter Binding Affinities at DAT and SERT of Substituted Cathinones and Cocaine^a^^,^^b^

	transfected HEK293 cells				
	monoamine uptake inhibition			transporter binding affinities	
compound	[^3^H]MPP^+^ uptake at hDAT	[^3^H]5-HT uptake at hSERT	hDAT/hSERT inhibition ratio	[^3^H]WIN 35,428 binding at hDAT	[^3^H] imipramine binding at hSERT
NEC	1.44 (±0.11)	>100	157	2.33 (±0.45)	>100
NEB	0.305 (±0.025)	51.20 (±1.51)	168	0.198 (±0.019)	90.07 (±6.13)
NEPD	0.091 (±0.018)	76.39 (±2.09)	844	0.042 (±0.007)	24.64 (±2.48)
NEH	0.073 (±0.013)	>100	1457	0.121 (±0.012)	35.94 (±8.51)
NEHP	0.251 (±0.024)	>100	426	0.107 (±0.018)	40.58 (±3.21)
α-PVP^c^	0.124 (±0.006)	>100	3222	0.019 (±0.002)	>100
Cocaine^c^	0.238 (±0.016)	2.01 (±0.28)	8	0.307 (±0.04)	0.56 (±0.04)

a For monoamine uptake inhibition assays, values are IC_50_ given as μM (mean ± SEM) and for transporter binding affinities assays, values are *K*_*i*_ given as μM (mean ± SEM) of four independent experiments carried out in triplicate. hDAT/hSERT inhibition ratios were also calculated as mentioned in the Methods section.

b hDAT/hSERT ratio = 1/DAT IC_50_:1/SERT IC_50_.

c Control compound.

The binding affinity constants (*K*_*i*_) of the tested drugs, assessed by their capacity to displace the corresponding radioligand binding to membranes obtained from HEK293 cells expressing DAT and SERT, are presented in Table 1, and the concentration–response curves are depicted in Figure 2 (panel C–D). NEB, NEPD, NEH, and NEHP have shown a greater affinity for DAT than cocaine, while NEC presented a lower affinity than cocaine to this transporter. On the other hand, all the cathinones under study showed a lower affinity for DAT than α-PVP. Particularly, an increased affinity for DAT has also been observed when increasing the length of the α-carbon chain from NEC to NEPD, although this affinity decreases with the addition of extra carbons. However, as it occurs with the DA uptake inhibition potency, NEH and NEHP possess a higher affinity for DAT than NEC and NEB. All compounds presented low affinity to SERT, which correlates with their low potency inhibiting 5-HT uptake. Since the potency of some synthetic cathinones to inhibit norepinephrine transporter (NET) is likely in the similar range as the potency to inhibit DAT,^24,45−47^ activity at NET might be more relevant than the weak activity at SERT and should be an important topic for future research. Indeed, NEH has been described as a potent NET blocker,^24^ which could be translated into adverse cardiovascular effects. Moreover, further studies are needed in order to investigate the interaction as well as the structure–activity relationship of these compounds with other monoaminergic transporters and receptors.

### 2.2 Role of the α-Carbon Chain Length in the Cytotoxic Potential

**Figure 3:**
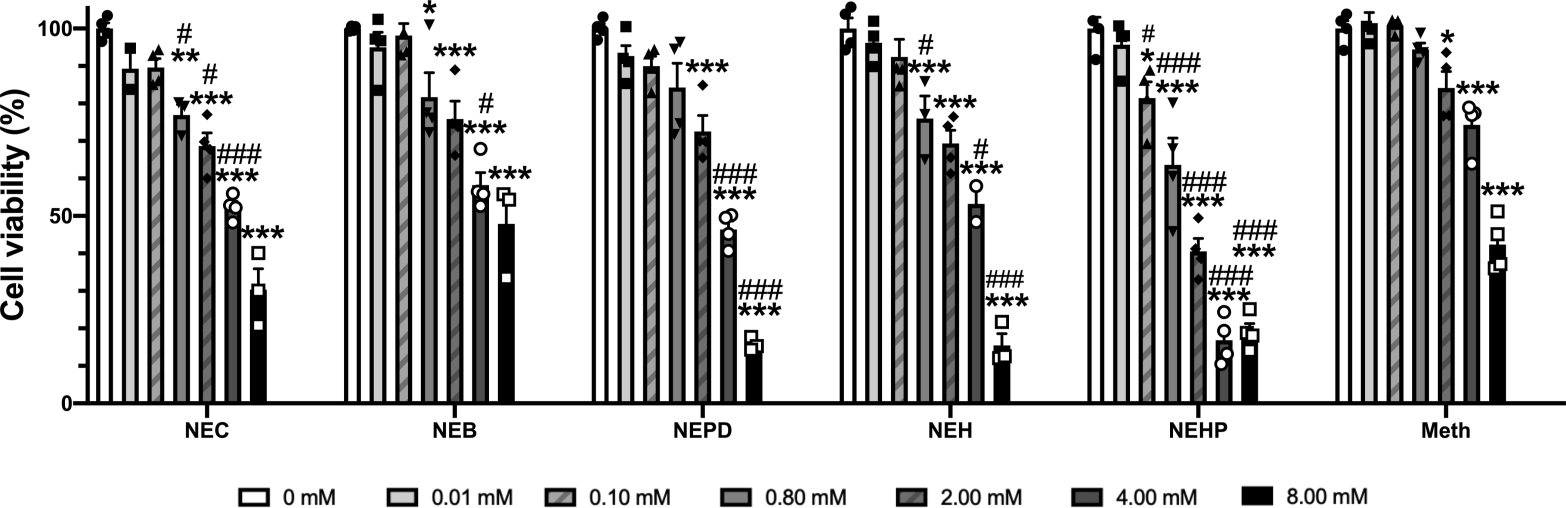
Evaluation of the cytotoxicity potential of synthetic cathinones in NGF-differentiated PC12 cells using the WST-8 assay. Results are expressed as a percentage (%) of cell viability (mean ± SEM) of 3–4 experiments carried out on triplicates. Tukey’s multiple-comparison test: *p < 0.05, **p < 0.01, and ***p < 0.001 vs the corresponding control (0 mM) group, #*p* < 0.05 and ###*p* < 0.001 vs the matching concentration of Meth.

The cytotoxicity potential of these cathinones has been examined with nerve growth factor (NGF)-differentiated pheochromocytoma cells (PC12) (see Figure 3). Two-way ANOVA of cell viability of PC12 cells after exposure to 0.01–8.00 mM, yielded the following results: drug variable: *F*_(5,115)_ = 38.65; *p* < 0.001; concentration variable: *F*_(6,115)_ = 269.2; *p* < 0.001; interaction variable: *F*_(30,115)_ = 4.815; *p* < 0.001. In the present study, all compounds decreased cell viability in a concentration-dependent manner, which is in accordance with the cytotoxicity reported for NEC and NEH in SH-SY5Y neuronal cells.^40,48^ NEHP, the cathinone with the longest α-carbon chain, showed cytotoxicity starting at 0.10 mM, a concentration that did not exhibit a cytotoxic effect for the rest of the studied cathinones. This suggests that increasing the length of the aliphatic side chain may increase cytotoxicity. This observation was also supported by the approximated LC_50_ values obtained (Table 2). In fact, Matsunaga and co-workers^39^ also observed a correlation of the chain elongation in α-pyrrolidinophenones with ROS production, considering that the cathinone-mediated monoamine inhibition and resultant monoamine depletion may be related to such toxicity. Moreover, Soares and co-workers^40^ have also demonstrated that the shortening of the lipophilic chain decreases the cytotoxicity of pentedrone and α-PVP. According to the same authors,^40^ these findings are in line with other studies showing a reduction in potency as DA uptake inhibitors when shortening the lipophilic chain.^28,29^ However, our study, in which molecules with longer carbon chains were tested (>3 carbon atoms), demonstrates that NEHP, despite not being the most potent compound inhibiting DA uptake, was capable of inducing cytotoxicity at lower concentrations than the other compounds tested. In this sense, elongation of the α-carbon chain is thought to increase lipophilicity, resulting in better penetration of the cell membrane. This is one of the most reasonable and plausible explanations for the structure–activity relationship for the cytotoxicity observed in our study.^49^ On the other hand, all the cathinones tested presented cytotoxicity starting at lower millimolar concentrations than methamphetamine did, suggesting that all the cathinones here studied present higher cytotoxicity potential than methamphetamine. However, the actual impact of these findings when translated to human consumption, where plasma levels reached are far lower, might not be relevant and should be further investigated. Nevertheless, we would like to point out that with this toxicity experiment, we are only assessing the direct toxic effects of the tested substances on the cells, which may imply specific mechanisms related to its interaction with the intracellular monoamines to more nonspecific effects. Therefore, the in vitro assessed toxicity does not rule out any other manifestations of toxicity, which may occur when administering the drug in vivo, where metabolism, integrated neurotransmission, and several physiological effects may play a role and would probably take place at lower plasma concentrations.

**Table 2:** Cytotoxicity Potential of Synthetic Cathinones in NGF-Differentiated PC12 Cells Assessed by the WST-8 Assay^a^

	PC12 cells
	cell viability assays
compound	LC50 (approximated)
NEC	3.97 (±0.39)
NEB	6.24 (±0.71)
NEPD	3.82 (±0.80)
NEH	3.28 (±0.44)
NEHP	1.26 (±0.25)
Meth^b^	8.73 (±1.24)

a Values are LC50 given as mM (mean ± SEM) of 3–4 independent experiments carried out in triplicate.

b Control compound.

### 2.3 Locomotor Activity and Anxiety-like Behavior Effects

**Figure 4:**
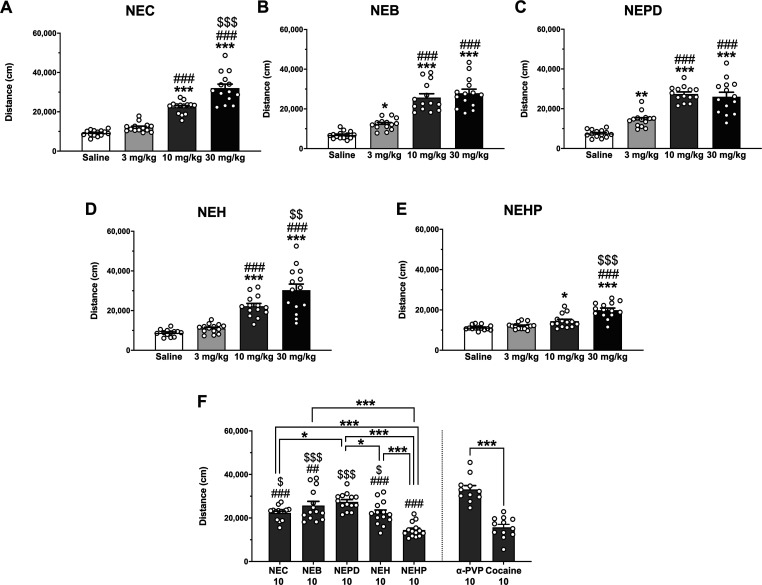
Effects of NEC (panel A), NEB (panel B), NEPD (panel C), NEH (panel D), and NEHP (panel E) on cumulative HLA in mice. Tukey’s multiple-comparison test: *p < 0.05, **p < 0.01, and ***p < 0.001 vs saline, ###*p* < 0.001 vs 3 mg/kg, $$*p* < 0.01, and $$$*p* < 0.001 vs 10 mg/kg. Panel F represents the effects of the NECs, α-PVP, and cocaine at 10 mg/kg on cumulative HLA in mice. Tukey’s multiple-comparison test: *p < 0.05, **p < 0.01, and ***p < 0.001, ##*p* < 0.01 and ###*p* < 0.001 vs α-PVP, $*p* < 0.05 and $$$*p* < 0.001 vs cocaine. Bars represent mean ± SEM of the total distance (cm) traveled in 60 min. *N* = 11–14/group.

It is well known that synthetic cathinones are able to induce psychostimulant effects; for a review, see ref (50). Therefore, we examined the horizontal locomotor activity (HLA) produced after an acute injection of the different *N*-ethyl cathinones. One-way ANOVA of the total distance traveled after drug administration revealed a significant effect of the variable dose for all the cathinones tested (NEC: *F*_(3,52)_ = 78.92; *p* < 0.001; NEB: *F*_(3,52)_ = 48.17; *p* < 0.001; NEPD: *F*_(3,52)_ = 49.04; *p* < 0.001; NEH: *F*_(3,51)_ = 32.47; *p* < 0.001; NEHP: *F*_(3,52)_ = 28.13; *p* < 0.001). The posthoc Tukey–Kramer test demonstrated a significant increase in HLA for all substances after 10 and 30 mg/kg injections compared to the saline group (see Figure 4, panels A–E). Although NEB and NEPD induced a ceiling effect at the highest dose tested (30 mg/kg), they were the only cathinones to have a significant effect after an acute dose of 3 mg/kg. On the other hand, NEC, NEH, and NEHP effects showed a dose–response relationship, which is in accordance with ref (23), which also reported an increase in locomotor activity following 10 and 25 mg/kg injections of NEH. Interestingly, although a significant increase in locomotor activity was observed after 10 and 30 mg/kg NEHP, its psychostimulant effect is remarkably lower than that produced by the other cathinones, suggesting a decrease of the HLA when increasing the length of the aliphatic side chain (see Figure 4, panel F). When analyzing the efficacy at the intermediate dose tested, one-way ANOVA yielded a significant effect of the variable drug (*F*_(6,86)_ = 21.40; *p* < 0.001). In fact, an inverted *U*-shape response can be observed among the *N*-ethyl cathinones tested. Particularly, NEPD, which possesses the same length of the alpha-carbon chain as α-PVP, induced a similar increase in locomotor activity at the medium dose tested (10 mg/kg). All the NECs tested, with the exception of NEHP, showed higher locomotion than cocaine at 10 mg/kg. Moreover, an increase of the α-alkyl-side-chain length from methyl to ethyl, propyl, and/or butyl induces an increase in locomotor activity, while the effect starts to decrease with the addition of extra carbons (Figure 4, panel F). These in vivo results are partially in accordance with the profiles obtained from DA uptake inhibition assays in which an increase of the α-alkyl-side-chain length from methyl to propyl also represents higher potency. However, some discrepancies between the in vitro versus in vivo results were observed. For instance, NEH seems to induce similar hyperlocomotion in comparison with NEC but is almost 20-fold more potent in inhibiting DA uptake. Such discrepancies might be explained by a different pharmacokinetic and metabolic profile in vivo as it has been stated by other authors when detecting differences in the pharmacological profile of other synthetic cathinones.^51^

**Figure 5:**
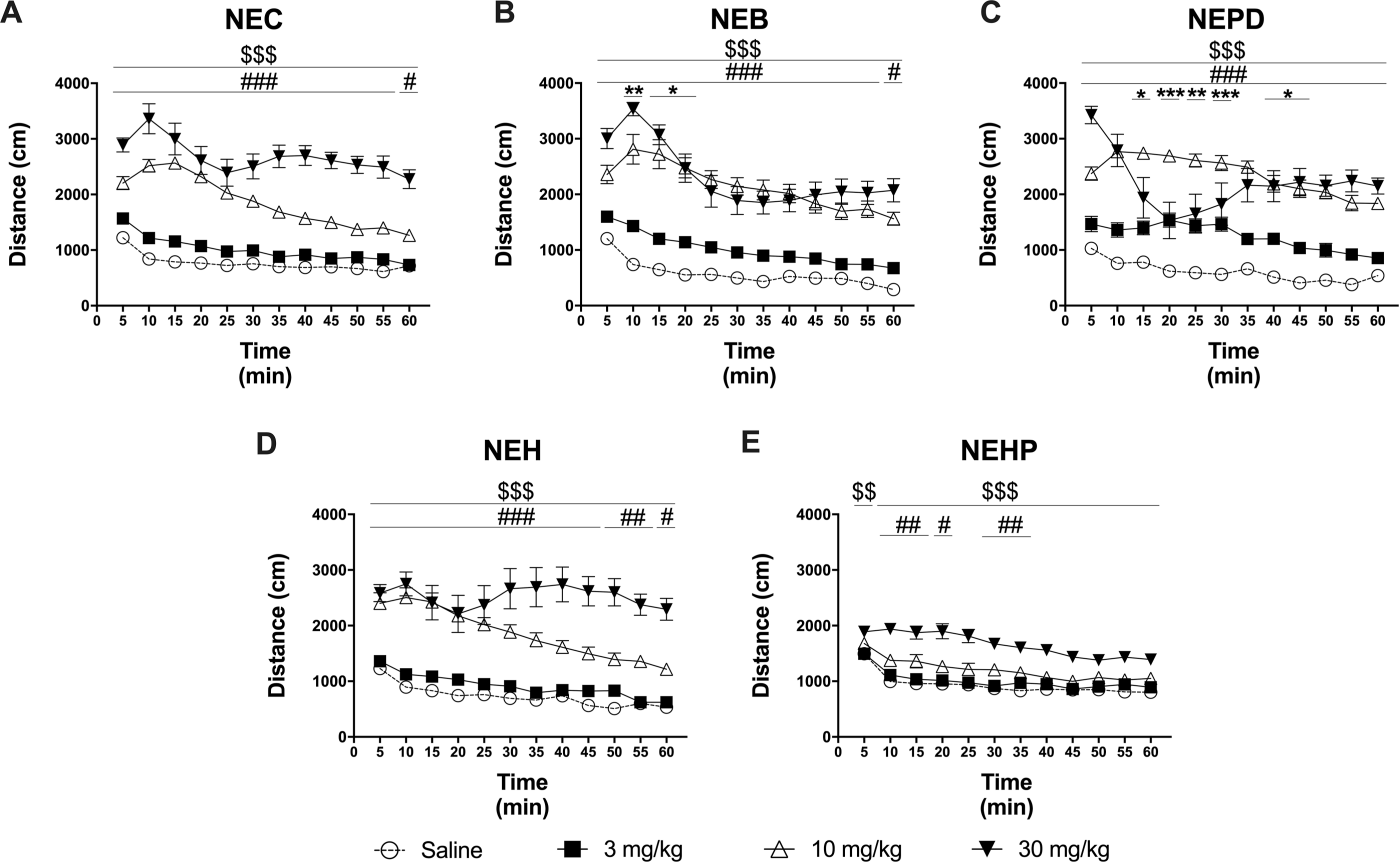
Time course profile of HLA induced by NEC (panel A), NEB (panel B), NEPD (panel C), NEH (panel D), and NEHP (panel E). Each time point represents the mean ± SEM of the distance (in cm) traveled in 5 min blocks. Only comparisons vs the corresponding saline group are shown for clarity purposes. Tukey’s multiple-comparison test: *p < 0.05, **p < 0.01 and ***p < 0.001, 3 mg/kg vs saline, #*p* < 0.05, ##*p* < 0.01, and ###*p* < 0.001 10 mg/kg vs saline, $$*p* < 0.01 and $$$*p* < 0.001 30 mg/kg vs saline. *N* = 13–14/group.

HLA profiles are depicted in Figure 5. Two-way ANOVA of repeated measures of the results yielded the following results: NEC: dose: *F*_(3,52)_ = 78.88; *p* < 0.001; time: *F*_(11,572)_ = 41.97; *p* < 0.001; interaction: *F*_(33,572)_ = 6.926; *p* < 0.001; NEB: dose: *F*_(3,52)_ = 48.43; *p* < 0.001; time: *F*_(11,572)_ = 56.88; *p* < 0.001; interaction: *F*_(33,572)_ = 6.346; *p* < 0.001; NEPD: dose: *F*_(3,52)_ = 49.04; *p* < 0.001; time: *F*_(11,572)_ = 19.02; *p* < 0.001; interaction: *F*_(33,572)_ = 6.041; *p* < 0.001; NEH: dose: *F*_(3,51)_ = 32.46; *p* < 0.001; time: *F*_(11,561)_ = 27.93; *p* < 0.001; interaction: *F*_(33,561)_ = 6.653; *p* < 0.001; NEHP: dose: *F*_(3,52)_ = 28.13; *p* < 0.001; time: *F*_(11,572)_ = 55.27; *p* < 0.001; interaction: *F*_(33,572)_ = 3.286; *p* < 0.001. A rapid onset effect (5–10 min) was observed after 10 and 30 mg/kg injections. As shown in Figure 5, the significant increase in the locomotor activity induced by NEB and NEPD at the lowest dose tested (3 mg/kg) lasted for 15 and 40 min, respectively. On the other hand, NEHP was the only cathinone whose effect at 10 mg/kg ended before 60 min. Regarding the highest dose tested (30 mg/kg), not only a ceiling effect was observed in the cumulative distance traveled induced by NEB and NEPD administrations (Figure 4B,C) but an abrupt decreasing slope after a high and initial hyperlocomotion was also observed (Figure 5B,C). Both events might be explained by the emergence of stereotypes, a common effect when injecting high doses of synthetic cathinones into rodents, which results in reduced hyperlocomotion.^50−54^

**Figure 6:**
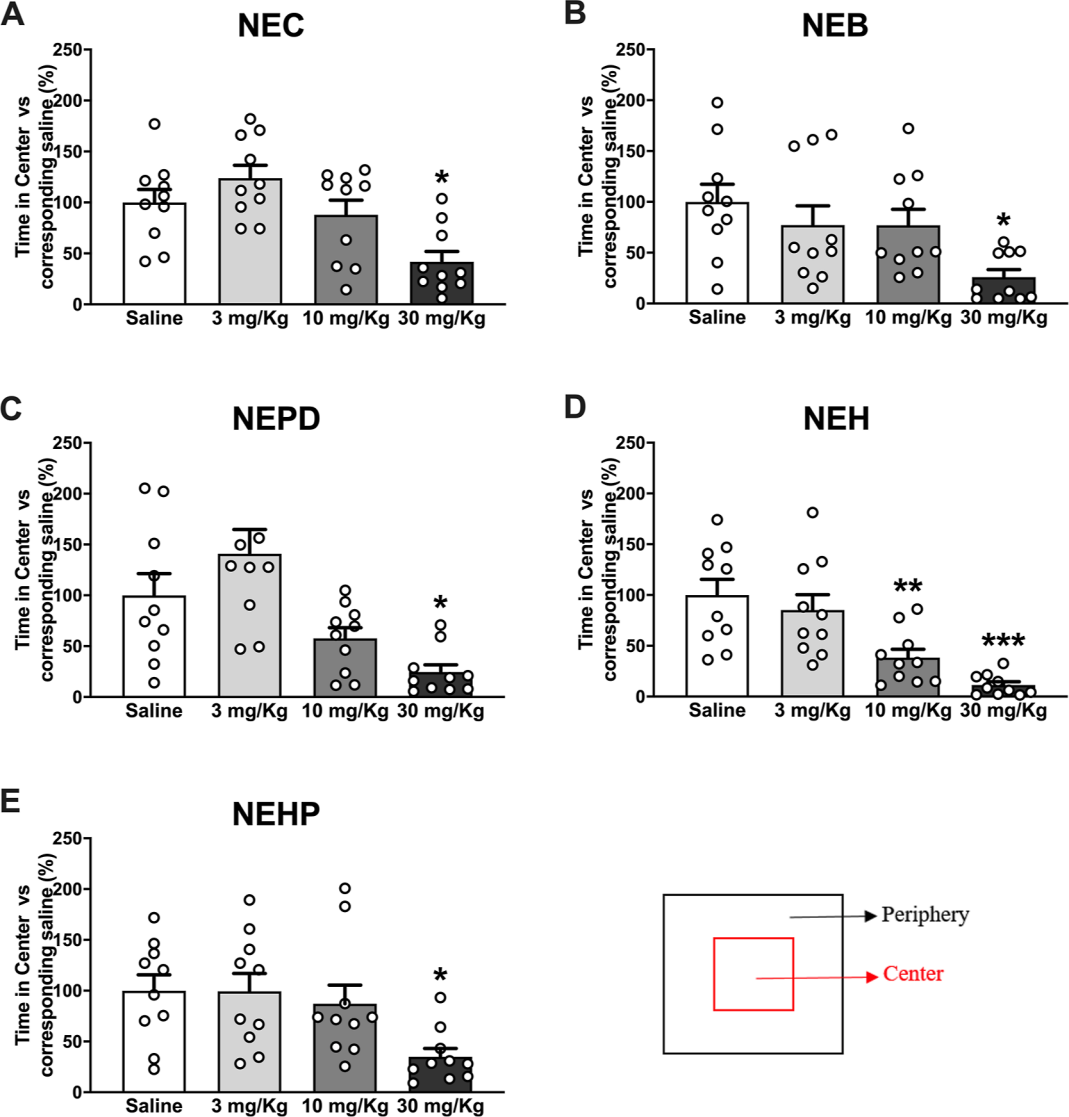
Effects of NEC (A), NEB (B), NEPD (C), NEH (D), and NEHP (E) on the OF test (anxiety-like behavior) in CD-1 mice. Bars represent mean ± SEM of time in the center, expressed as a percentage vs its corresponding saline. Tukey’s multiple-comparison test: *p < 0.05 and **p < 0.01 vs saline. *N* = 10/group.

One of the methods widely used to study whether a compound may induce anxiety-like behaviors is the open-field (OF) test, in which a decrease in the time that the animals spend in the center (or an increase in the time that the animals spend in the periphery) of the arena is related to an anxiogenic effect.^55^ In our study, the time spent in the center of the OF arena after an i.p injection of saline or the corresponding drug dose is presented in Figure 6. One-way ANOVA of the results yielded a significant effect of the variable dose for all the synthetic cathinones tested: NEC: *F*_(3,36)_ = 7.603; *p* < 0.001; NEB: *F*_(3,36)_ = 4.097; *p* < 0.05; NEPD: *F*_(3,36)_ = 8.591; *p* < 0.001; NEH: *F*_(3,36)_ = 12.31; *p* < 0.001; NEHP: *F*_(3,48)_ = 3.974; *p* < 0.05. All the cathinones presented a significant decrease in the time spent in the center of the arena at the highest dose tested, suggesting an acute anxiogenic effect after a 30 mg/kg injection. Additionally, NEH was the only cathinone tested able to induce acute anxiogenic effects at the medium dose tested (10 mg/kg). None of the tested compounds presented anxiogenic effects after a 3 mg/kg injection.

On the other hand, several authors have suggested that dopaminergic mechanisms seem to be involved in the generation of anxiety, which would explain the presence of anxiogenic effects of some well-known DAT blockers and DA releasers such as cocaine and methamphetamine.^56−59^ This may also apply to the tested compounds in this study since they are able to inhibit DA uptake and induce anxiety-like behaviors in mice. However, our study only shows initial evidence of such anxiety-like behavior, but we must point out that more experiments are needed in order to corroborate the anxiogenic effect of the substances tested.

### 2.4 Rewarding Effects

**Figure 7:**
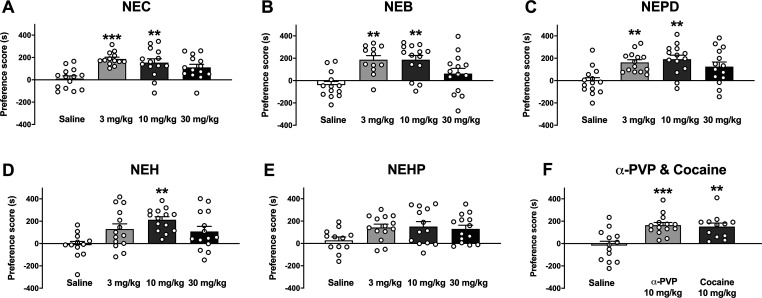
Effects of NEC (panel A), NEB (panel B), NEPD (panel C), NEH (panel D), NEHP (panel E), and α-PVP and cocaine (panel F) on the CPP test in mice. Bars represent the mean ± SEM of the preference score (difference between the time spent in the drug-paired compartment on the test day and the preconditioning day). Tukey’s multiple-comparison test: **p < 0.01 and ***p < 0.001 vs saline. *N* = 12–14/group.

The rewarding effects of NEC, NEB, NEPD, NEH, NEHP, α-PVP, and cocaine were studied using the conditioned place preference (CPP) paradigm. Seven animals were withdrawn from the experiments due to an initial preference for one of the compartments (>70% of the total session time). On the test day, one-way ANOVA of the results yielded a significant effect of dose for all the synthetic cathinones tested (NEC: *F*_(3,51)_ = 7.499; *p* < 0.001; NEB: *F*_(3,49)_ = 7.176; *p* < 0.001; NEPD: *F*_(3,52)_ = 5.819; *p* < 0.01; NEH: *F*_(3,50)_ = 3.912; *p* < 0.05; NEHP: *F*_(3,50)_ = 2.463; *p* > 0.05; α-PVP and cocaine: *F*_(2,36)_ = 10.48; *p* < 0.001). In this study, the rewarding properties of NEC and NEB are reported for the first time. Previous studies have demonstrated the ability of NEH to induce significant CPP after 4 and 16 mg/kg subcutaneous injections.^48^ As shown in Figure 7, our results demonstrate that NEC, NEB, NEPD, and NEH produce rewarding effects in mice at the medium dose tested. Although NEHP repeated administration induced an increase in the preference score, this did not reach significance at any dose tested. This finding may correlate with the lowest psychostimulant effect also observed in vivo for NEHP. On the other hand, the anxiety-like behavior induced by all the cathinones tested at the dose of 30 mg/kg may have a significant impact on the balance between reward and aversive behavior since a lack of significant rewarding effects at the same dose was also observed, suggesting that a 30 mg/kg dose of the cathinones tested can be equivalent to a high dose with unpleasant effects.

As expected, repeated administration of α-PVP and cocaine (10 mg/kg) induced rewarding effects in the CPP paradigm. Moreover, NEC, NEB, and NEPD were also able to induce a significant preference score in the CPP paradigm at the lowest and medium dose tested (3 and 10 mg/kg), while NEH did only at 10 mg/kg. On the other hand, NEHP did not induce a significant rewarding effect at any dose tested. However, it must be taken into account that the CPP paradigm has particular limitations when talking about potency or effectiveness, and therefore when trying to correlate such effect with other parameters (i.e., IC_50_ values, EC_50_, *K*_*i*_, etc...). In fact, often, an all-or-nothing effect is observed, with a threshold dose above which CPP is observed, albeit not with a dose-dependent increase in effect magnitude, resulting in a more qualitative than quantitative model.^60^ Actually, despite the increasing knowledge about the neuropharmacology of synthetic cathinones, many questions remain unanswered, including the poorly understood role of nontransporter sites of action, drug pharmacokinetics, and drug metabolism, which may explain some of the unexpected effects (for a review, see ref (50)). Moreover, further studies of the reinforcing effects of the synthetic cathinones tested in this study, including self-administration, discriminative stimulus, and intracranial self-stimulation experiments, will be necessary.

In summary, our results show that the length of the α-carbon chain in *N*-ethyl-substituted cathinones plays an important role in their pharmacological and toxicological properties. An increase in the aliphatic side-chain length from methyl to propyl has translated into higher DAT inhibition potency and affinity. The IC_50_ values at inhibiting DA uptake have stayed in the same order of magnitude when increasing from propyl to butyl, but the addition of an extra carbon has resulted in a decrease in the potential to inhibit this transporter. All of the cathinones tested presented little or null activity at the SERT and are therefore more DAT-selective than cocaine, which might indicate high abuse liability. Moreover, all the cathinones tested have shown higher cytotoxicity than methamphetamine. Particularly, NEHP, the cathinone with the longest side chain, shows cytotoxic effects at lower concentrations than the analogues tested, although a low psychostimulant and no significant rewarding effect was observed in vivo for this cathinone. On the other hand, an increase in the locomotor activity of mice has been observed for all the tested compounds, which correlates with their potency inhibiting DA uptake in vitro. Furthermore, the rewarding properties of NEC and NEB are reported for the first time. Finally, given the recent increase in the number of NPS detected, especially synthetic cathinones, our findings may provide guidance as to which novel synthetic cathinones might pose serious risks to public health and should be considered for future scheduling and control measures.

## 3 Methods

### 3.1 Subjects

All animal care and experimental protocols are approved by the Animal Ethics Committee of the University of Barcelona under the supervision of the Autonomous Government of Catalonia and are in accordance and compliance with the guidelines of the European Community Council (2010/63/EU), as amended by Regulation (EU) 2019/1010, and the ARRIVE guidelines for reporting experiments involving animals.^61^ Male Swiss CD-1 mice (Janvier, Le Genest, France) weighing 30–35 g (6–8 weeks old), housed in temperature-controlled conditions (22 ± 1 °C) under a 12 h light/dark cycle and with free access to food and drinking water (standard laboratory diet, Panlab SL, Barcelona, Spain), were randomly assigned to an experimental group. Efforts were made to minimize animal use and suffering.

### 3.2 Drugs and Materials

*N*-Ethyl cathinones were synthesized in a racemic form as hydrochloride salts as described in Section 3.3. Solutions for injection were prepared daily in an isotonic saline solution (0.9% NaCl, pH 7.4). Cocaine and methamphetamine hydrochloride were generously provided by the Spanish National Institute of Toxicology and Dr. Riera laboratory from Parc Científic de Barcelona, respectively. α-PVP·HCl was synthesized as described in ref (6). Cell culture media [Dulbecco’s modified Eagle’s medium (DMEM) high-glucose] was purchased from Sigma-Aldrich. [^3^H]1-Methyl-4-phenylpyridinium ([^3^H]MPP^+^) was supplied by American Radiolabeled Chemicals (St. Louis, USA). [^3^H]5-HT, [^3^H]imipramine, and [^3^H]WIN35,428 were purchased from PerkinElmer Inc. (Boston, MA, USA). The NGF was supplied by Upstate Biotechnology (Lake Placid, NY). The CCK-8 cell counting kit was purchased from Vazyme Biotech Co., Ltd. All other reagents were of analytical grade and purchased from several commercial sources.

### 3.3 Chemistry

**Scheme 1:**
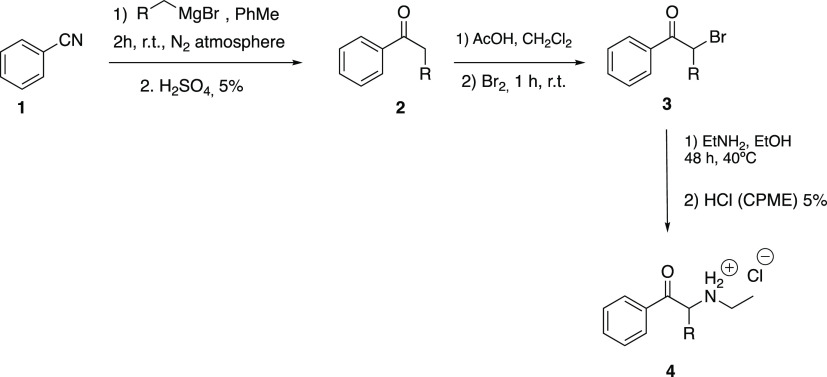
Synthesis of *N*-Ethyl Cathinones

The synthesis of the synthetic cathinones was carried out following the procedure described in ref (47). Benzonitrile (**1**) was subjected to reaction with the corresponding Grignard reagent in anhydrous conditions, followed by acidic hydrolysis, to achieve the intermediate ketone (**2**). α-Halogenation was achieved by the addition of bromine (Br_2_) to a solution of **2** in dichloromethane (CH_2_Cl_2_), with catalytic amounts of glacial acetic acid (AcOH). Reaction with ethylamine (EtNH_2_) gave the synthetic cathinone (**3**), which was crystallized as a hydrochloride salt. The identification of the cathinone was assessed by thin-layer chromatography, proton and carbon nuclear magnetic resonance (^1^H NMR and ^13^C NMR), infrared spectroscopy, and liquid chromatography–mass spectrometry Scheme 1. For characterization, see the Supporting Information.

### 3.4 Uptake Inhibition and Transporter Binding Assays in HEK293 Cells

#### 3.4.1 Cell Culture and Membrane Preparation

Human embryonic kidney cells (HEK293) stably transfected with the human DAT and SERT were used for both the uptake inhibition and transporter binding assays.^6^ Cells were cultured in DMEM supplemented with heat-inactivated 10% fetal bovine serum (FBS), 1 μg/mL streptomycin, and 100 U/mL penicillin at 37 °C in a 5% CO_2_ humidified atmosphere. Geneticin (G418; 50 μg/mL) was added to maintain the selection process. Upon approximately 80% confluence, cells were washed with 5 mL of phosphate-buffered saline (PBS) and 1 mL of trypsin/EDTA was added. After 3 min, 9 mL of DMEM was added to stop cell trypsinization. If necessary, centrifugation was performed to pellet the cells. Cells were then resuspended, counted, and seeded onto poly-d-lysine coated (24 h/prior to the experiment) 96-well plates at a density of 0.36 million cells per well for the uptake inhibition assays.

For membrane preparation, cells were harvested from 80 to 90% confluent dishes. HEK293 cells were washed with ice-cold phosphate-buffered saline (PBS), mechanically detached from the dish using a plastic scraper, and pelleted by centrifugation (400*g* for 10 min at 4 °C). The pellet was resuspended in HME buffer (20 mM HEPES NaOH, 1 mM EDTA, 2 mM MgCl_2_; pH 7.4), subjected to two freeze–thaw cycles in liquid nitrogen and homogenized through sonication at 4 °C. Membranes were then collected by centrifugation (40,000*g* for 30 min at 4 °C) and resuspended in an appropriate volume of HME buffer. The different membrane preparations aliquots were kept at −80 °C. Protein concentration was determined using the Bio-Rad Protein Reagent (Bio-Rad Laboratories, Hercules, CA).

#### 3.4.2 Uptake Inhibition Assays

Assays were performed as previously described.^8^ On the test day, the medium was removed from the cell culture 96-well plates and immediately replaced with 200 μL of Krebs-HEPES-buffer (KHB; 10 mM HEPES, 120 mM NaCl, 3 mM KCl, 2 mM CaCl_2_·2H_2_O, 2 mM MgCl_2_·6H_2_O supplemented with 20 mM d-glucose; pH 7.3). To ensure equilibrated conditions, cells were incubated with different concentrations of the drugs in KHB at a final volume of 50 μL/well for 5 min (preincubation). The preincubation solution was removed, and cells were incubated with the tritiated compounds, 0.02 μM [^3^H]MPP^+^ for hDAT and 0.1 μM [^3^H]5-HT for hSERT, together with various concentrations of the test drugs in KHB. The uptake incubation times were 3 min for hDAT and 1 min for hSERT. The reaction was stopped by rapid removal of the incubation solution and washout with ice-cold KHB. Cells were lysed with 1% sodium dodecyl sulfate, and lysates were subsequently transferred to vials containing scintillation fluid. Radioactivity was quantified with a beta-scintillation counter (PerkinElmer, Waltham, MA, USA). The uptake in the absence of the test drugs was normalized to 100%, and the uptake in the presence of various concentrations of cathinones was expressed as a percentage thereof. Nonspecific uptake was assessed in parallel samples containing cocaine 100 μM for DAT-expressing HEK293 cells and paroxetine 3 μM for SERT-expressing HEK293 cells. Four independent experiments carried out on triplicates were performed.

#### 3.4.3 Transporter Binding Assays

Binding assays were performed as described.^6^ The cathinones under study were dissolved in binding buffer (120 mM NaCl, 3 mM KCl, 10 μM ZnCl_2_, 2 mM MgCl_2_, and 20 mM Tris pH 7.4 for hDAT, and 120 mM NaCl, 3 mM KCl, 2 mM MgCl_2_, 1 mM EDTA, and 20 mM Tris pH 7.4 for hSERT) at a range of concentrations from 0.1 nM to 1 M. The membrane preparations that overexpressed the transporter hDAT or hSERT were incubated with radiolabeled ligands at concentrations close to or equal to *K*_d_, and ligand displacement by the test drugs was measured in duplicate. The binding assays were performed in tubes containing 25 μL of [^3^H]WIN35,428 (*K*_d_ = 12 nM; B_max_ = 6.75 pmol/mg^62^) for hDAT, at a final concentration of 10 nM, or [^3^H]imipramine (*K*_d_ = 4.5 nM; *B*_max_ = 15 pmol/mg^62^) for hSERT, at a final concentration of 3 nM, diluted in reaction buffer, 15 μg of membranes in 100 μL of reaction buffer, and 125 μL of the tested drug dilution. Nonspecific binding was determined in the presence of high concentrations of cocaine (100 μM) and paroxetine (3 μM) as these drugs are able to fully displace [^3^H]WIN35,428 and [^3^H]imipramine binding at hDAT and hSERT, respectively. This nonspecific binding also allows subtracting from the total binding values of the binding to other components such as membrane lipids or microfiber filters. Incubation was performed for 1 h at 22 °C. Binding reactions are stopped by rapid filtration of the membranes through GF/C glass microfiber filters presoaked with 0.5% polyethyleneimine and rapid washing with ice-cold wash buffer (120 mM NaCl, 2 mM MgCl_2_, 10 mM Tris, and 100 μM ZnCl_2_ for hDAT, and 120 nM NaCl, 2 mM MgCl_2_, and 10 mM Tris, for hSERT). Thereafter, the filters are placed into vials, a scintillation cocktail is added and trapped radioactivity is quantified by liquid scintillation counting. Specific binding of each drug to the transporter was defined as the difference between total binding (binding buffer in absence of the drug) and nonspecific binding. For each cathinone, four independent experiments carried out on duplicates were performed.

### 3.5 Cytotoxicity Assays in PC12 Cells

#### 3.5.1 PC12 Cell Culture and Differentiation

Pheochromocytoma cells (PC12) cells were cultured in collagen-coated dishes in DMEM supplemented with heat-inactivated 5% FBS, 10% horse serum, 10 mM HEPES, 2 mM glutamine, 25 UI/mL penicillin, and 25 μg/mL streptomycin (maintenance medium) and grown at 37 °C in a humidified 5% CO_2_ atmosphere. Cell differentiation was performed as described^54^ with minor modifications. In brief, cells were seeded (250.000 cells/well) onto 96-well plates in the maintenance medium, and after 24 h, the medium was changed to a differentiation medium containing 50 ng/mL NGF. After 24 h, neurite outgrowth was already apparent.

#### 3.5.2 Cell Viability by the WST-8 Assay

Treatments with different concentrations of the drug in DMEM were performed 48 h after cell differentiation. 10 μL of the tested drug was added per well, in triplicate, and cells were incubated for 24 h at 37 °C in a humidified 5% CO_2_ atmosphere. Thereafter, the medium was removed and immediately replaced with a maintenance medium. 10 μL of the CCK-8 Cell Counting Kit, based on 2-(2-methoxy-4-nitrophenyl)-3-(4-nitrophenyl)-5-(2,4-disulfophenyl)-2*H*-tetrazolium, monosodium salt (WST-8), was added per well and was incubated for 2 h at 37 °C in a humidified 5% CO_2_ atmosphere. This method has been defined as a good alternative to the 3-(4,5-dimethylthiazol-2-yl)-2,5-diphenyltetrazoliumbromide (MTT) assay.^63^ Optical density was measured at 450 nm, using a microplate reader.

### 3.6 Horizontal Locomotor Activity

In the habituation phase, which lasted for two consecutive days, mice received an intraperitoneal (i.p.) saline injection and were immediately placed into a black Plexiglass OF arena (25 × 25 × 40 cm) under low-light conditions and white noise for 30 min. On the test day, the HLA was measured as described.^8^ In short, the animals received an i.p. injection of saline (5 mL/kg) or different doses (3, 10, or 30 mg/kg) of the corresponding drug (NEC, NEB, NEPD, NEH, or NEHP), or the reference compounds cocaine and α-PVP (10 mg/kg) and was immediately placed in the OF arena with the same conditions of light and noise. HLA was video-monitored for 1 h and a specific tracking software (Smart 3.0 Panlab, Barcelona, Spain) was used to measure their total traveled distance (in cm). The doses were chosen according to the psychostimulant effect induced by structurally related synthetic cathinones published in previous studies by our research group.^8^

### 3.7 OF Test: Center Versus Periphery

The anxiety-like effects of the test compounds were assessed through an OF test as described.^64^ Animals were placed individually in the center of an open-field arena (25 cm length × 25 cm width × 40 cm height), and the time spent, in seconds, in the center (8 × 8 cm) or the periphery of the arena was monitored for 60 min (Smart 3.0 Panlab, Barcelona, Spain).

### 3.8 Conditioned Place Preference

The potential of the studied compounds to induce reward was determined using a place conditioning paradigm, as described.^6^ For this experiment, an apparatus with two distinct compartments, with differences in tactile and visual cues, communicated by a central corridor, has been used. In brief, CPP was performed in three phases: preconditioning test, conditioning, and postconditioning test. During the preconditioning phase (day 0), mice were placed in the middle of the corridor and had free access to both compartments for 15 min. The time spent in each compartment was recorded and monitored using a specific tracking software (Smart 3.0 Panlab, Barcelona, Spain). During the conditioning phase (day 1–day 4; two sessions per day separated by a 5 h period), the access to the corridor was closed, and mice received an i.p. injection of the corresponding cathinone or cocaine and were immediately placed into one of the compartments for 20 min. In the alternative session, mice received a saline i.p. injection and were placed for 20 min in the other compartment. Mice in the control group received a saline injection in every session. The compartment and session in which mice received the drug were randomized. On the test day (postconditioning phase), the same conditions as in the preconditioning phase were applied. A preference score was calculated as the difference between the time spent in the drug-paired compartment in the postconditioning test minus the time spent in the preconditioning phase.

### 3.9 Data Analysis

For in vitro assays, data were normalized with 100% defined as the mean of the technical replicates in the control group and expressed as mean ± SEM. Nonlinear regression was used to fit the different competition curves. Data were plotted and best fitted to a sigmoidal dose–response curve from which an IC_50_ or LC_50_ value was obtained. Transporter ratios were calculated as (1/DAT IC_50_: 1/SERT IC_50_). Although IC_50_ values at inhibiting 5-HT uptake for some compounds tested are expressed as >100 μM for clarity purposes, DAT/SERT ratios were calculated with the original raw data. The Cheng–Prusoff equation was used to calculate *K*_i_ (affinity): *K*_i_ = EC_50_/(1 + [radioligand concentration/*K*_d_]).^65^ The sample size for behavioral experiments was determined using GPower software. One-way or two-way ANOVA, and subsequent Tukey’s posthoc test, conducted only if F was significant, was used to determine the effects of cathinones on cytotoxicity, HLA, OF, and CPP experiments. The α error probability was set at 0.05 (*p* < 0.05). The exact size group for the behavioral experiments is shown in the corresponding figure legends. All statistic calculations were carried out using GraphPad Prism (GraphPad software, San Diego, CA, USA).

## Supporting Information Available

^1^H-NMR, ^13^C-NMR, IR, and MS spectra for all compounds and concentration–effect curves on cell viability, expressed in percentage, of the tested cathinones in NGF-differentiated PC12 cells using the WST-8 assay (PDF)

## Abbreviations

WST-8: 2-(2-methoxy-4-nitrophenyl)-3-(4-nitrophenyl)-5-(2,4-disulfophenyl)-2*H*-tetrazolium, monosodium salt
AcOH: acetic acid
α-PVP: alpha-pyrrolidinovalerophenone
^13^C-NMR: carbon nuclear magnetic resonance
CPP: conditioned place preference
CH_2_Cl_2_: dichloromethane
DA: dopamine
DAT: dopamine transporter
EtNH_2_: ethylamine
HLA: horizontal locomotor activity
HEK293: human embryonic kidney cells
IR: infrared spectroscopy
LC/MS: liquid chromatography–mass spectrometry
Meth: methamphetamine
NEB: *N*-ethyl-buphedrone
NEC: *N*-ethyl-cathinone
NEHP: *N*-ethyl-heptedrone
NEH: *N*-ethyl-hexedrone
NEPD: *N*-ethyl-pentedrone
NGF: nerve growth factor
NPS: new psychoactive substances
OF: open field
PC12: pheochromocytoma cells
^1^H-NMR: proton nuclear magnetic resonance
5-HT: serotonin
SERT: serotonin transporter
TLC: thin-layer chromatography
NET: norepinephrine transporter
