# Supplementary material for: Structure–Activity Relationship of N-Ethyl-Hexedrone Analogues: Role of the α-Carbon Side-Chain Length in the Mechanism of Action, Cytotoxicity, and Behavioral Effects in Mice
Source: ACS Chem Neurosci. 2023 Feb 3;14(4):787–99. doi: 10.1021/acschemneuro.2c00772 (PMC9936538; doi:10.1021/acschemneuro.2c00772)
Supplement: Supplementary file 1 — cn2c00772_si_001.pdf [file cn2c00772_si_001.pdf]

# Supporting Information

## Structure-activity relationship of N-ethyl-hexedrone analogs: role of the α-carbon side chain length in the mechanism of action, citotoxicity and behavioral effects in mice.

*Núria Nadal-Gratacós [1,2], Edwin Ríos-Rodríguez [2], David Pubill [1], Xavier Batllori [2], Jorge Camarasa [1], Elena Escubedo [1], Xavier Berzosa [2, *], Raúl López-Arnau [1, *]*

*[1] Department of Pharmacology, Toxicology and Therapeutic Chemistry, Pharmacology Section and Institute of Biomedicine (IBUB), Faculty of Pharmacy, University of Barcelona, 08028 Barcelona, Spain.*

*[2] Pharmaceutical Chemistry Group (GQF), IQS School of Engineering, Universitat Ramon Llull, 08017 Barcelona, Spain.*

**Corresponding authors: raullopezarnau@ub.edu, xavier.berzosa@iqs.url.edu*

*Spectral data of synthesized compounds:*

***Ethcathinone***:
$^{1}$H-NMR (400 MHz, Chloroform-*d*) *δ*: 10.68 (s, 1H), 9.01 (s, 1H), 7.97 (dd, *J* = 8.5, 1.3 Hz, 2H), 7.68 – 7.63 (m, 1H), 7.52 (t, *J* = 7.7 Hz, 2H), 5.03 (dq, *J* = 7.4, 3.4 Hz, 1H), 3.23 (ddt, *J* = 11.7, 8.4, 4.2 Hz, 1H), 3.17 – 3.09 (m, 1H), 1.82 (d, *J* = 7.2 Hz, 3H), 1.54 (t, *J* = 7.2 Hz, 3H).
$^{13}$C NMR (100 MHz, Chloroform-*d*) δ 194.92 , 135.05 , 133.00 , 129.43 , 128.99 , 58.10 , 42.36 , 16.93 , 11.98 .
IR (KBr), ν max: 2934, 2805, 2686, 1693, 1438, 1237, 977, 793 cm$^{-1}$.
m/z: 177 (M+)
***NEB***:
$^{1}$H-NMR (400 MHz, Chloroform-*d*) *δ*: 10.84 (s, 1H), 8.56 (s, 1H), 8.00 (dd, *J* = 8.4, 1.2 Hz, 2H), 7.70 – 7.65 (m, 1H), 7.54 (t, *J* = 7.7 Hz, 2H), 5.06 (dq, *J* = 8.6, 4.4, 2.8 Hz, 1H), 3.26 (ddt, *J* = 12.0, 7.7, 3.9 Hz, 1H), 3.10 (ddt, *J* = 15.3, 7.6, 3.5 Hz, 1H), 2.50 – 2.40 (m, 1H), 2.30 – 2.20 (m, 1H), 1.55 (t, *J* = 7.3 Hz, 3H), 0.99 (t, *J* = 7.6 Hz, 3H).
$^{13}$C NMR (100 MHz, Chloroform-d) *δ 194.93 , 135.24 , 133.96 , 129.49 , 128.92 , 62.46 , 43.05 , 24.25 , 11.97 , 9.20 .*
IR (KBr), ν max: 2939, 2688, 2513, 1682, 1448, 1226, 976, 704 cm$^{-1}$.
m/z: 191 (M+)
***NEPD***:
$^{1}$H-NMR (400 MHz, Chloroform-d) *δ*(ppm): 11.33 (s, 1H), 8.07 (s, 1H), 7.99 (d, *J* = 7.4 Hz, 2H), 7.70 (t, *J* = 7.4 Hz, 1H), 7.56 (t, *J* = 7.7 Hz, 2H), 5.01 – 4.94 (m, 1H), 3.29 – 3.20 (m, 1H), 3.08 – 2.99 (m, 1H), 2.38 – 2.28 (m, 1H), 2.23 – 2.13 (m, 1H), 1.54 (t, *J* = 7.1 Hz, 3H), 1.50 – 1.42 (m, 1H), 1.38 – 1.30 (m, 1H), 0.88 (t, *J* = 7.2 Hz, 3H).
$^{13}$C NMR (100 MHz, DMSO-*d*6) δ 196.33 , 134.77 , 134.02 , 129.19 , 128.74 , 60.43 , 41.15 , 31.76 , 17.17 , 13.66 , 11.13 .
IR (KBr) *ν*max (cm-1): 3428, 2960, 1687, 1449, 1237, 1002, 709 cm$^{-1}$.

m/z : 206 (M+).

***NEH***:

$^{1}$H-NMR (400 MHz, Chloroform-*d)* $\delta$: 11.35 (s, 1H), 7.99 (dd, *J* = 8.4, 1.2 Hz, 2H), 7.73 – 7.67 (m, 1H), 7.56 (t, *J* = 7.7 Hz, 2H), 4.98 (t, *J* = 4.9 Hz, 1H), 3.32 – 3.19 (m, 1H), 3.10 – 2.98 (m, 1H), 2.41 – 2.30 (m, 1H), 2.27 – 2.14 (m, 1H), 1.54 (t, *J* = 7.3 Hz, 3H), 1.44 – 1.37 (m, 1H), 1.34 – 1.18 (m, 3H), 0.80 (t, *J* = 7.1 Hz, 3H).

$^{13}$C NMR (100 MHz, Chloroform-d) $\delta$: 195.21 , 135.31 , 133.94 , 129.52 , 128.93 , 61.68 , 43.10 , 30.71 , 26.71 , 22.56 , 13.70 , 12.02 .

IR (KBr), v max: 2950, 2736, 1690, a448, a228, 945, 700 cm$^{-1}$.

m/z: 219 (M+).

**NEHP**:

$^{1}$H-NMR (400 MHz, DMSO-*d6)* $\delta$: 11.36 (s, 1H), 7.99 (d, *J* = 7.4 Hz, 2H), 7.70 (t, *J* = 7.4 Hz, 1H), 7.56 (t, *J* = 7.7 Hz, 2H), 4.98 (d, *J* = 6.7 Hz, 1H), 3.26 (d, *J* = 11.5 Hz, 1H), 3.03 (d, *J* = 6.2 Hz, 1H), 2.34 (d, *J* = 15.6 Hz, 1H), 2.19 (d, *J* = 2.7 Hz, 1H), 1.54 (t, *J* = 7.2 Hz, 3H), 1.45 – 1.39 (m, 1H), 1.26 (s, 1H), 1.24 – 1.16 (m, 4H), 0.77 (t, *J* = 7.0 Hz, 3H).

$^{13}$C NMR (100 MHz, DMSO-*d6*) $\delta$: 195.29, 135.05, 134.29, 129.41, 128.92, 77.48, 77.16, 76.84, 61.47, 42.75, 31.57, 30.89, 24.57, 22.24, 13.97, 11.88.

IR (KBr), v max: 2938, 2858, 2681, 1684, 1449, 1247, 959, 699 cm$^{-1}$.

m/z: 233 (M+).

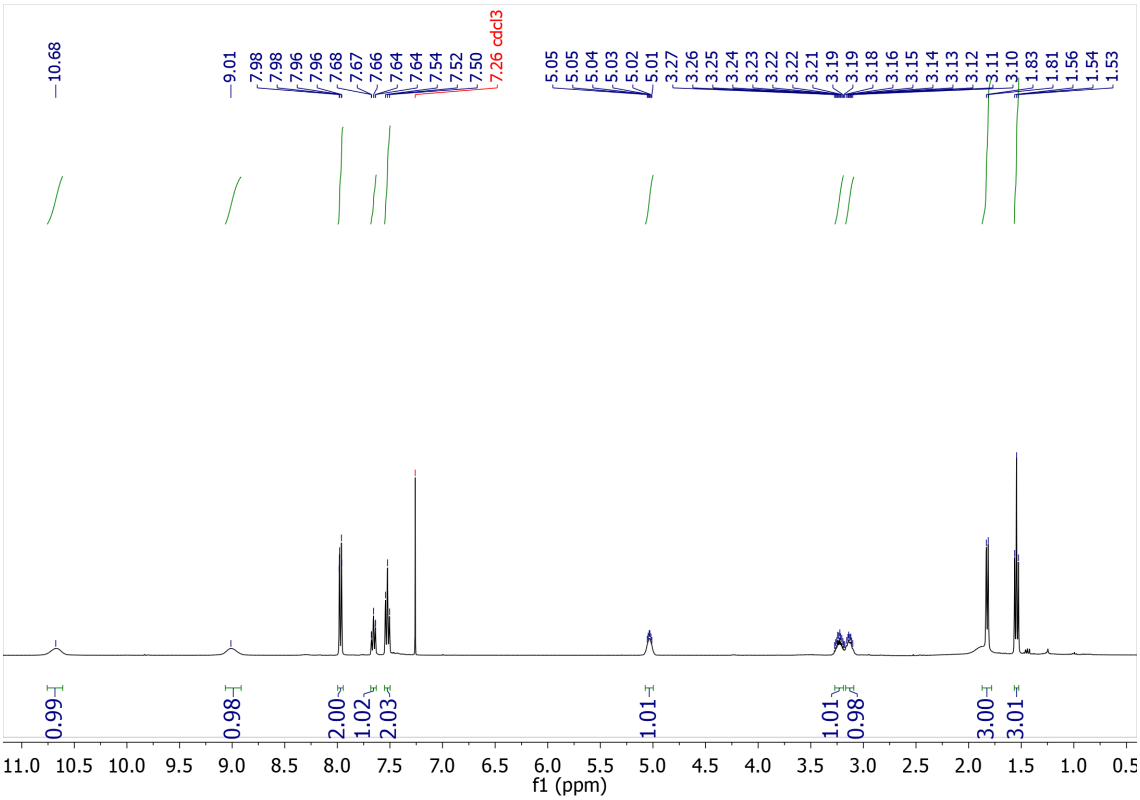


**Figure S1**: $^{1}$H-NMR spectrum of NEC.

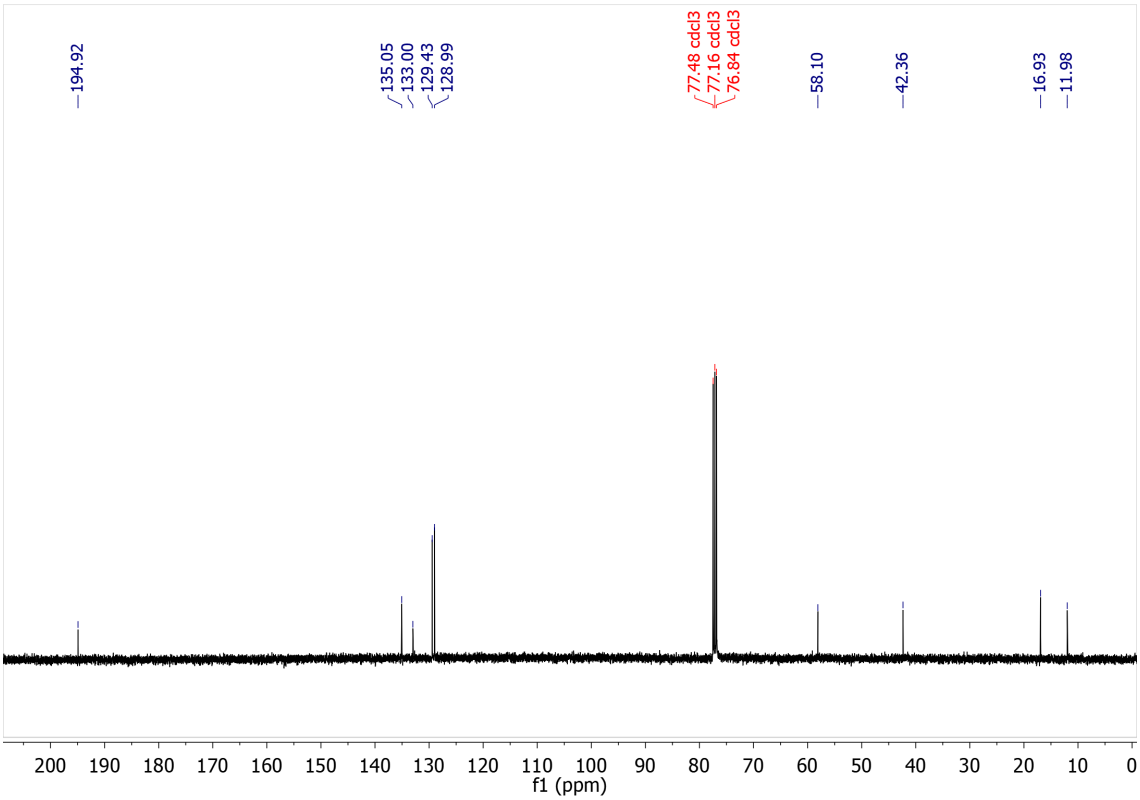


**Figure S2**: $^{13}$C-NMR spectrum of NEC.

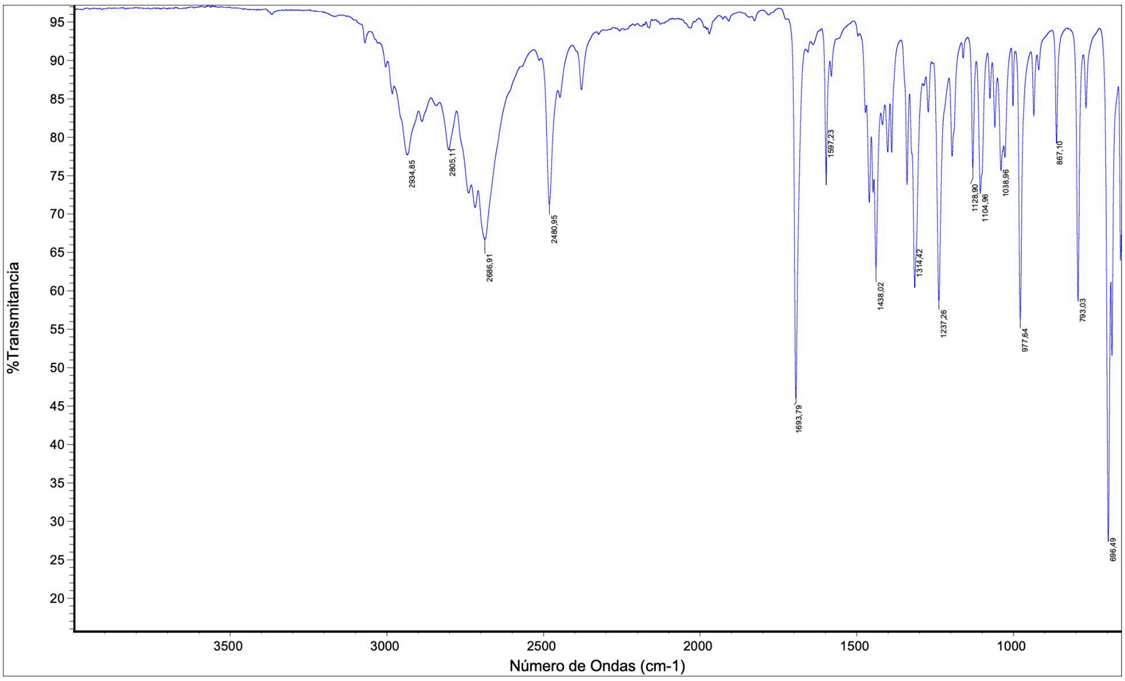


**Figure S3**: IR spectrum of NEC.

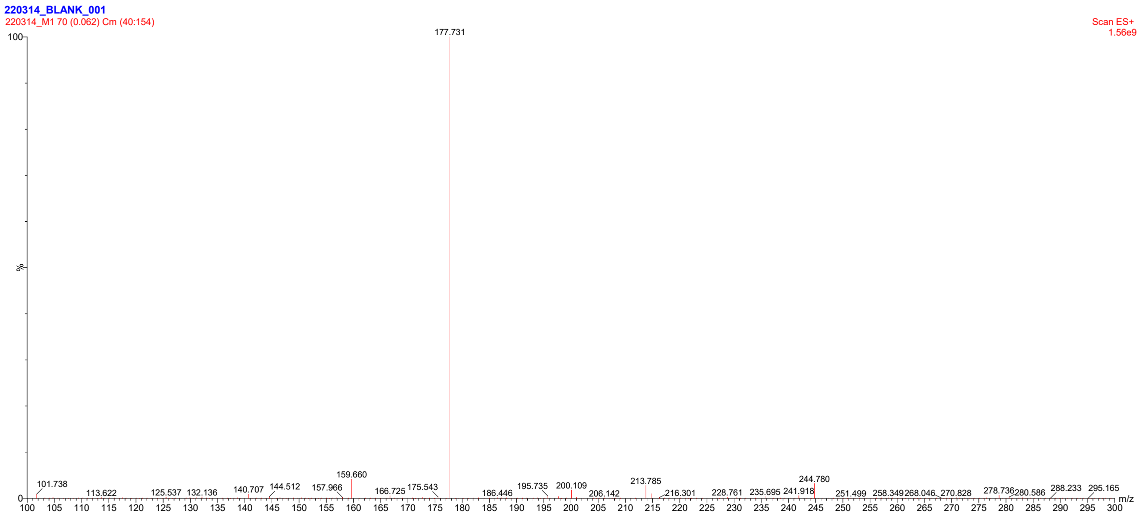

**Figure S4**: MS spectrum of NEC.

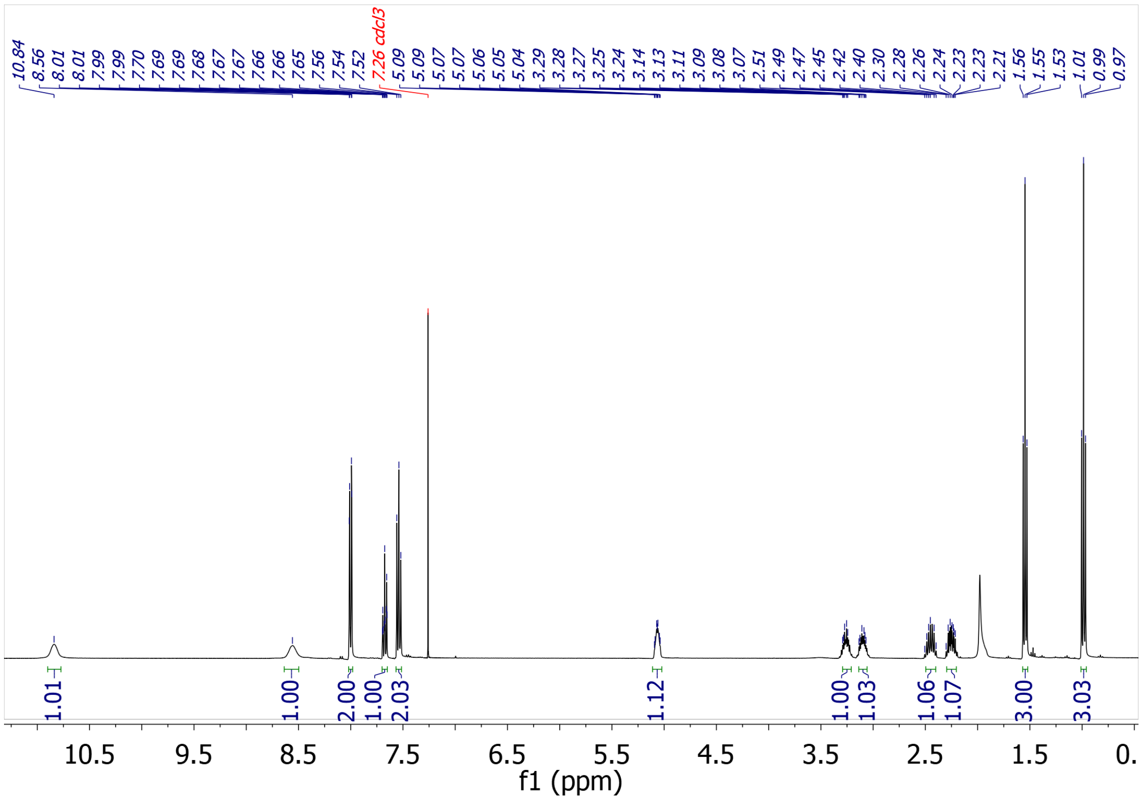

**Figure S5**: $^{1}$H-NMR spectrum of NEB.

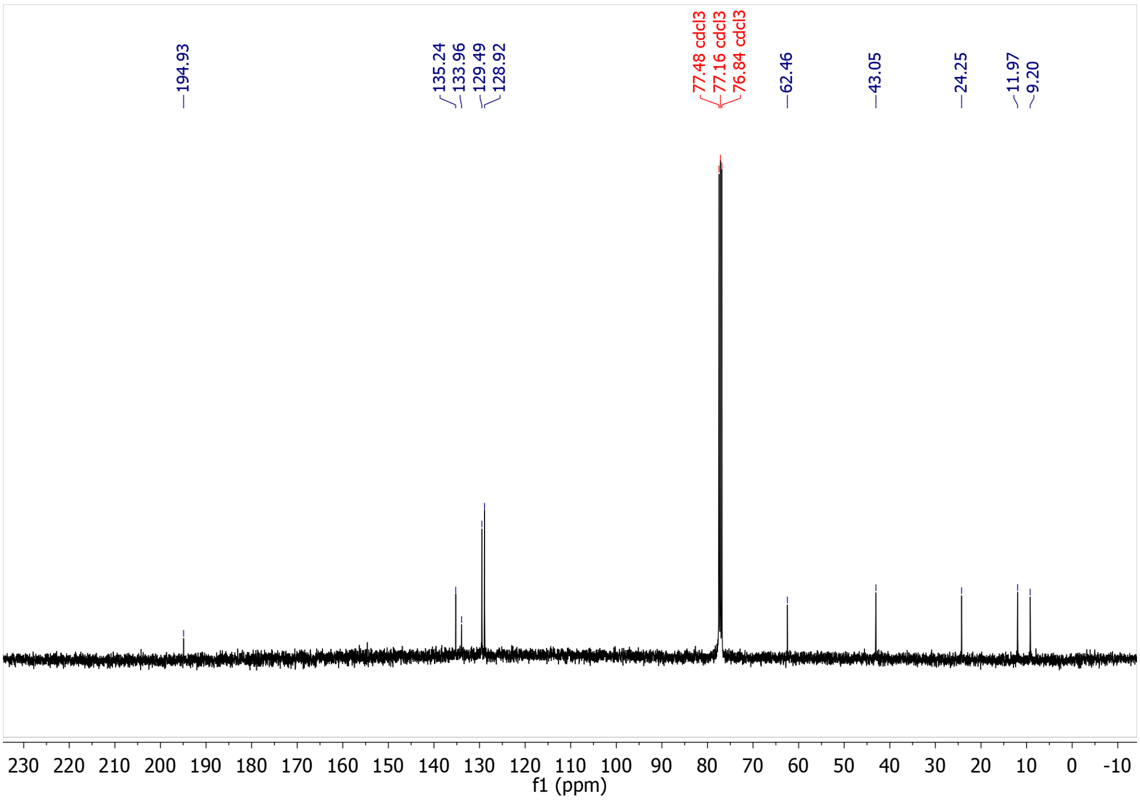


**Figure S6**: $^{13}$C-NMR spectrum of NEB.

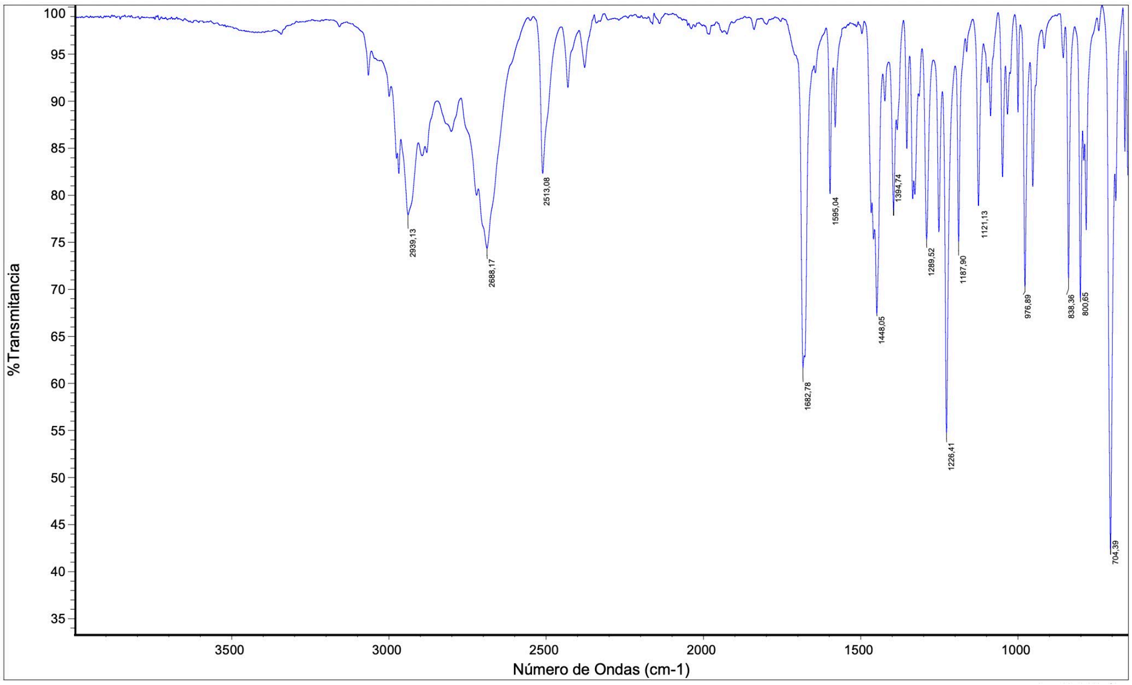


**Figure S7**: IR spectrum of NEB.

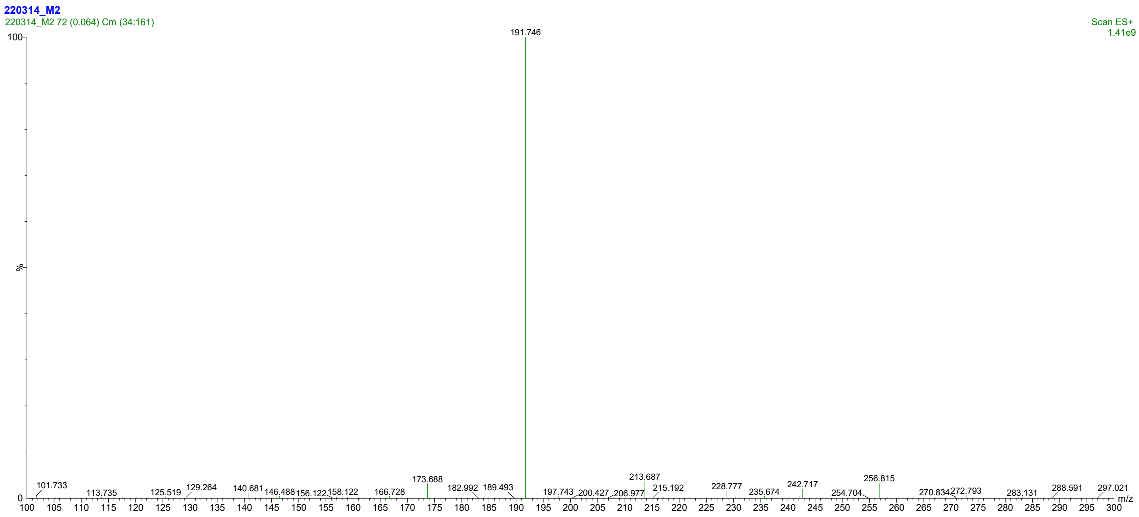

**Figure S8**: MS spectrum of NEB.

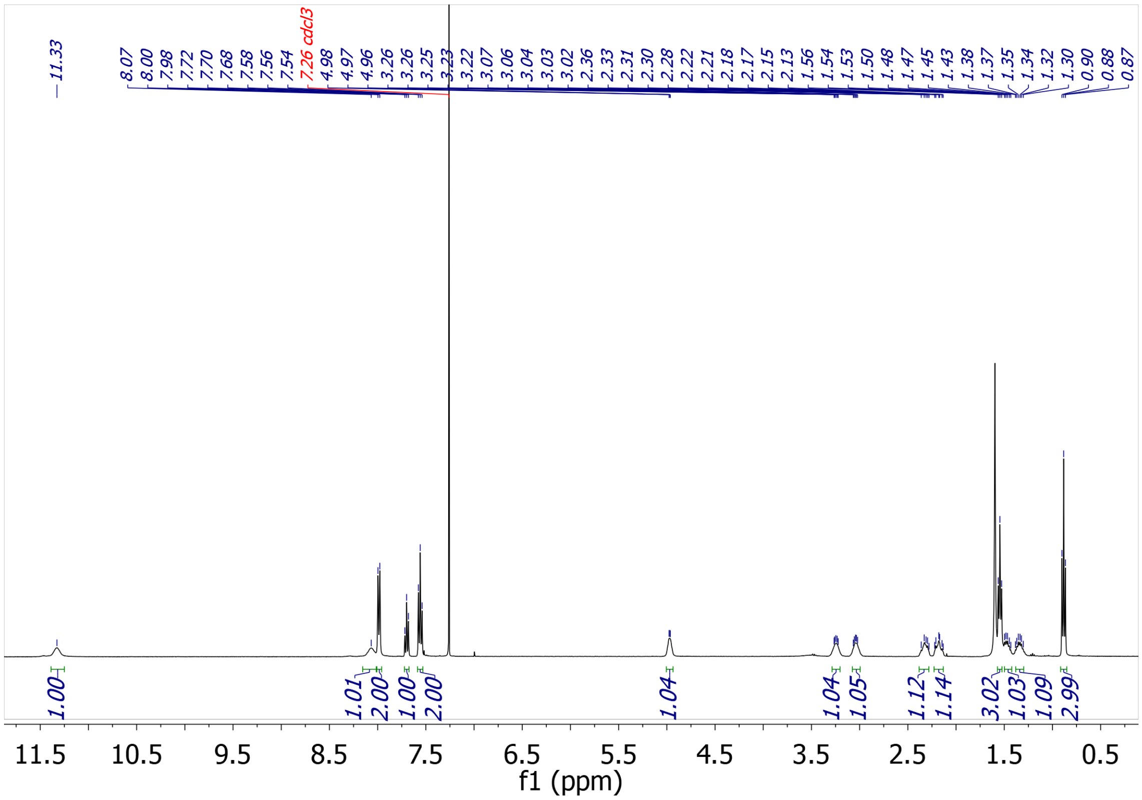

**Figure S9**: $^{1}$H-NMR spectrum of NEPD.

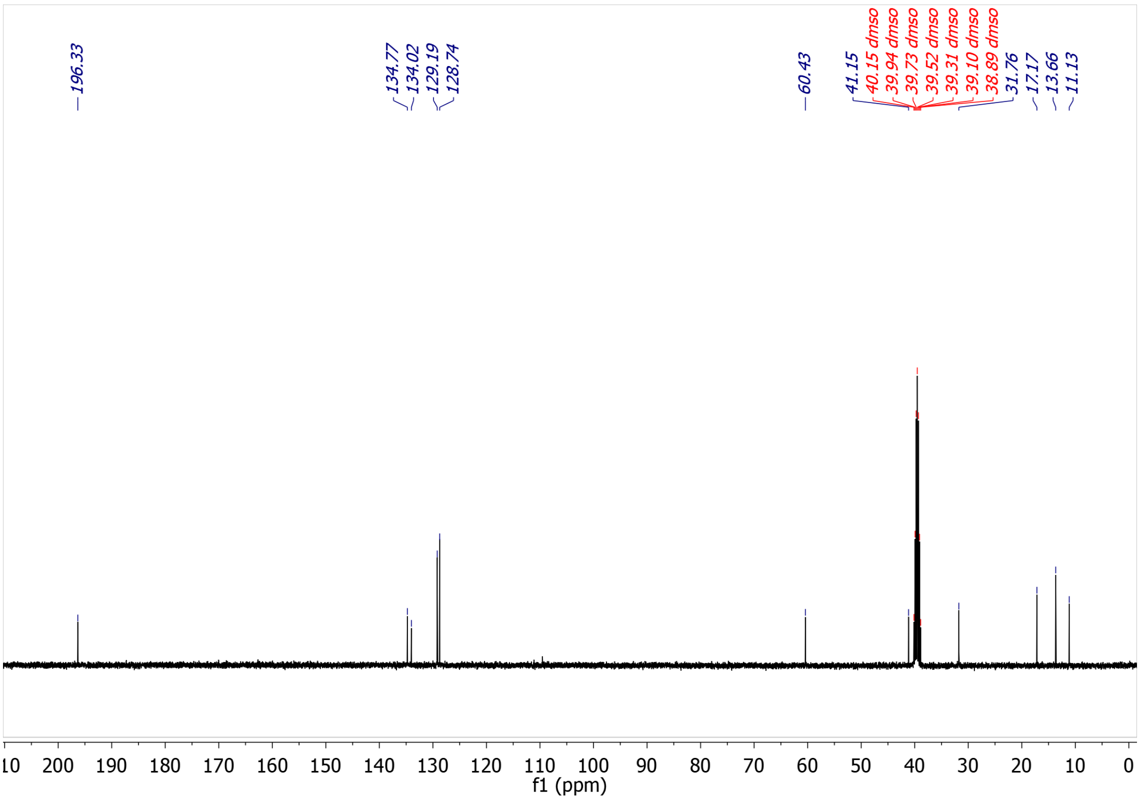


**Figure S10**: $^{13}$C-NMR spectrum of NEPD.

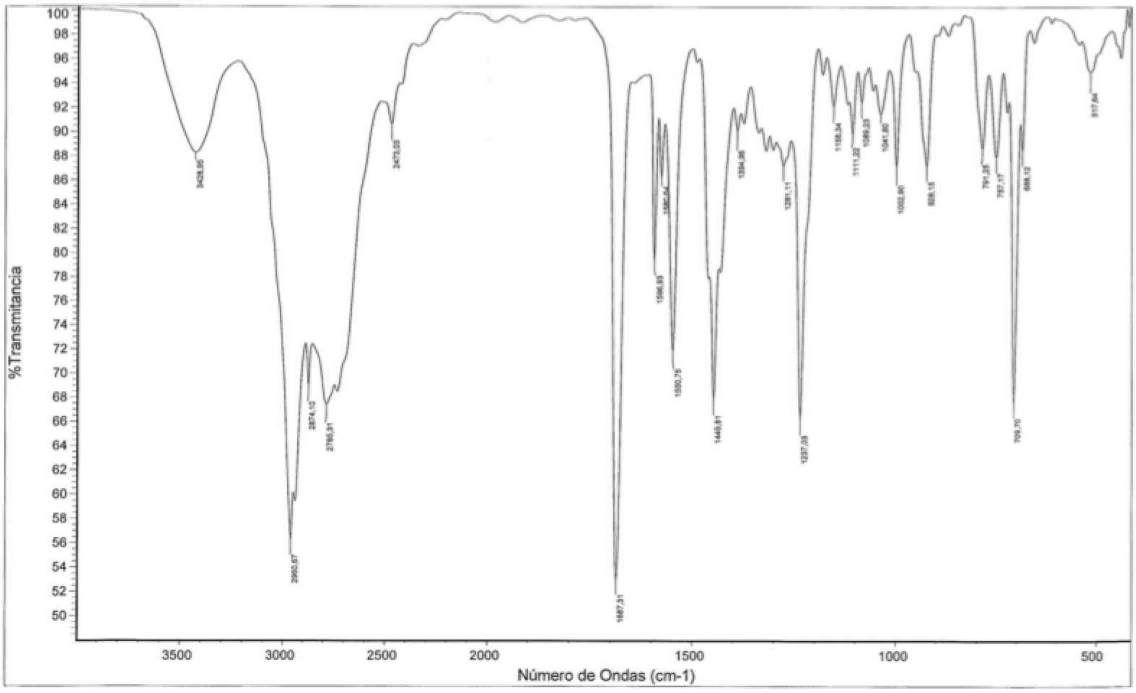


**Figure S11**: IR spectrum of NEPD.

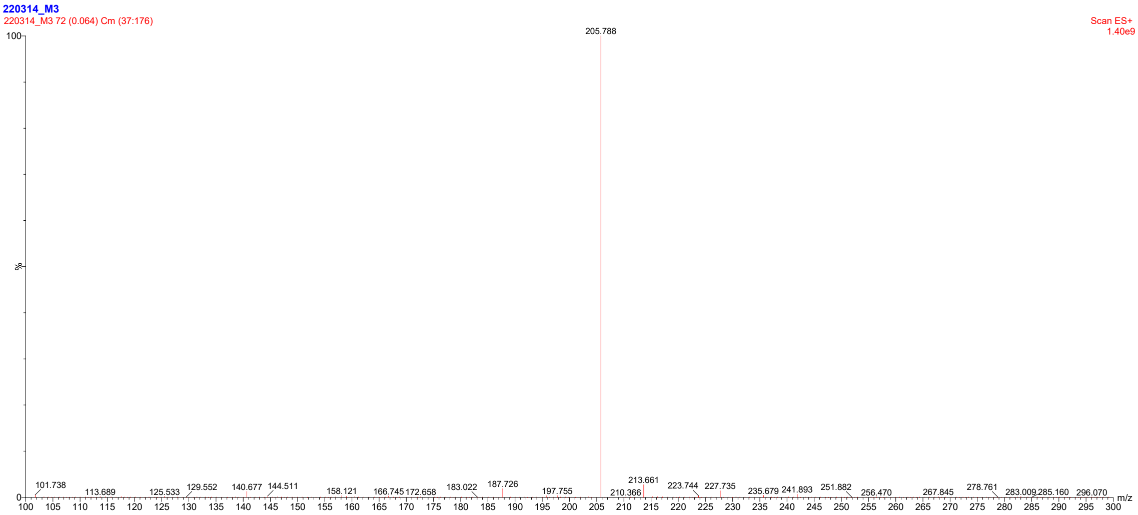


**Figure S12**: MS spectrum of NEPD.

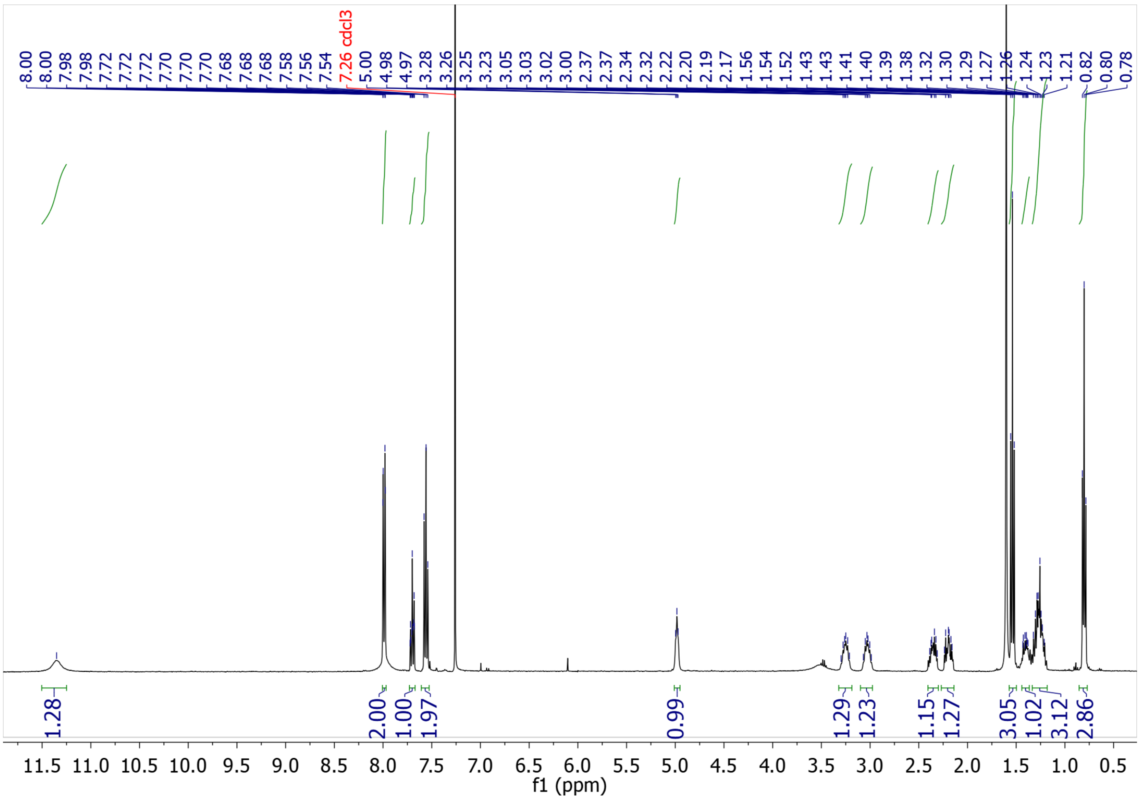


**Figure S13**: $^{1}$H-NMR spectrum of NEH.

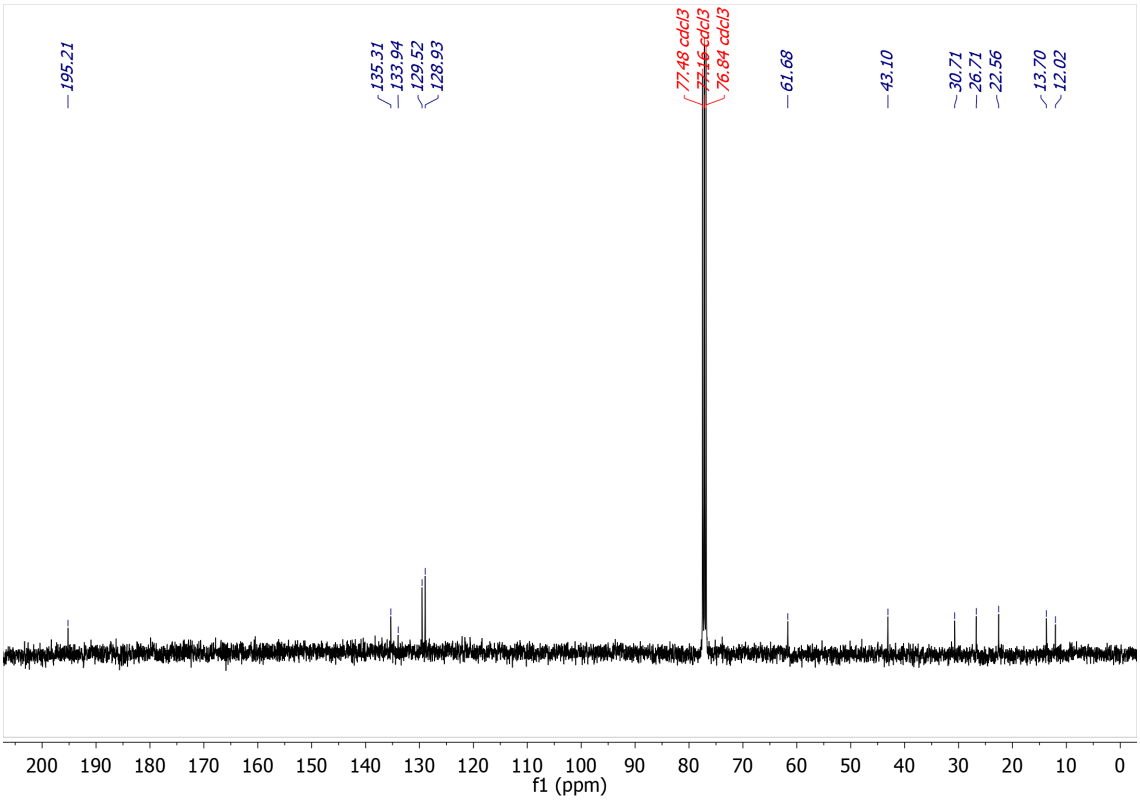


**Figure S14**: $^{13}$C-NMR spectrum of NEH.

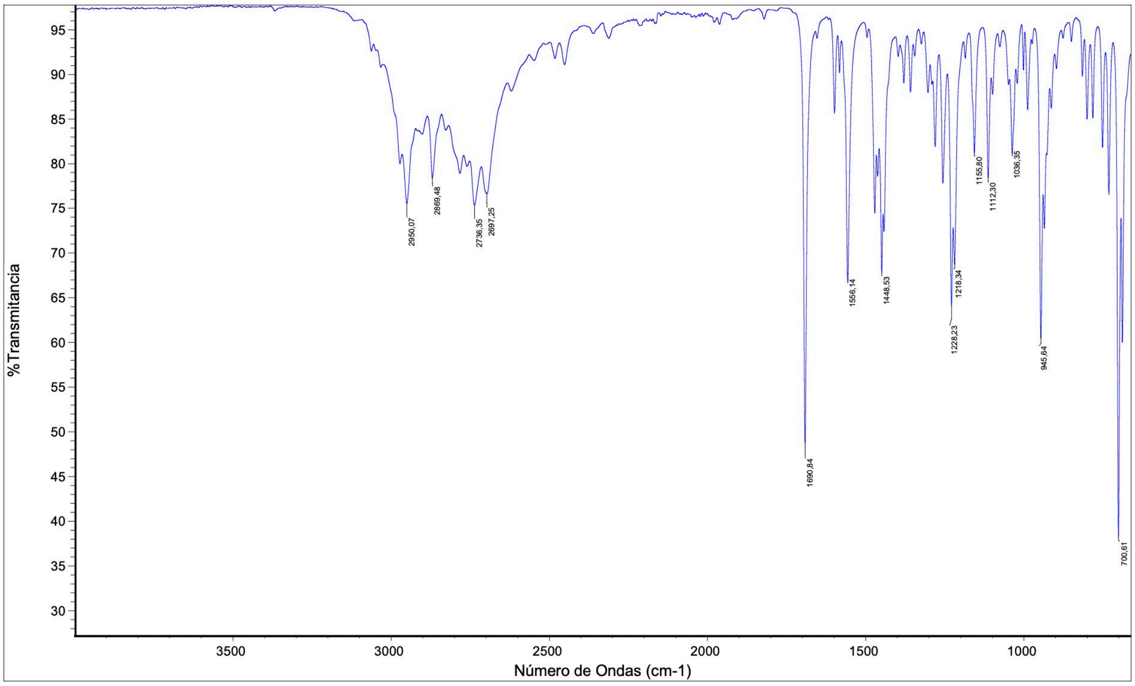


**Figure S15**: IR spectrum of NEH.

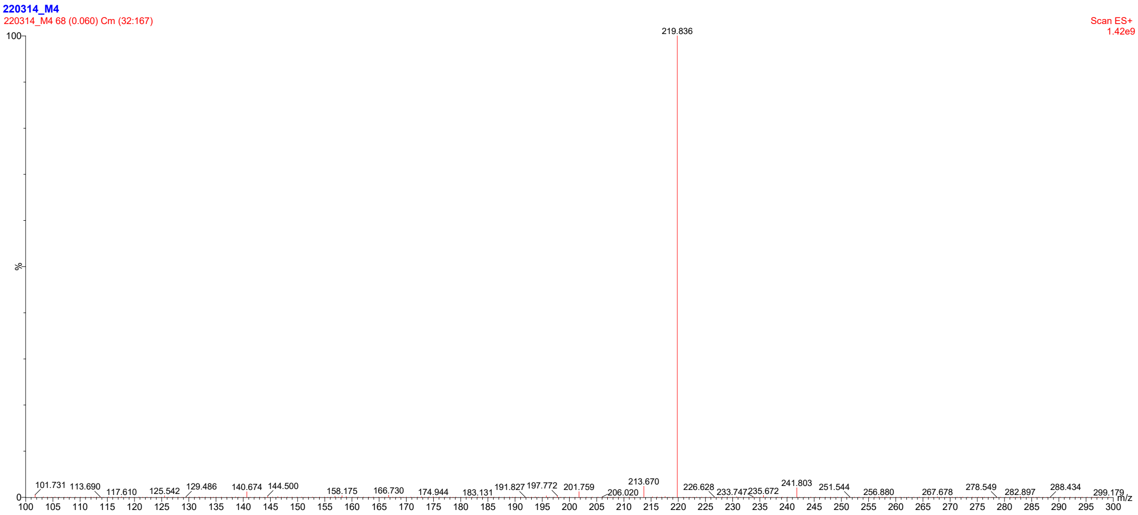


**Figure S16**: MS spectrum of NEH.

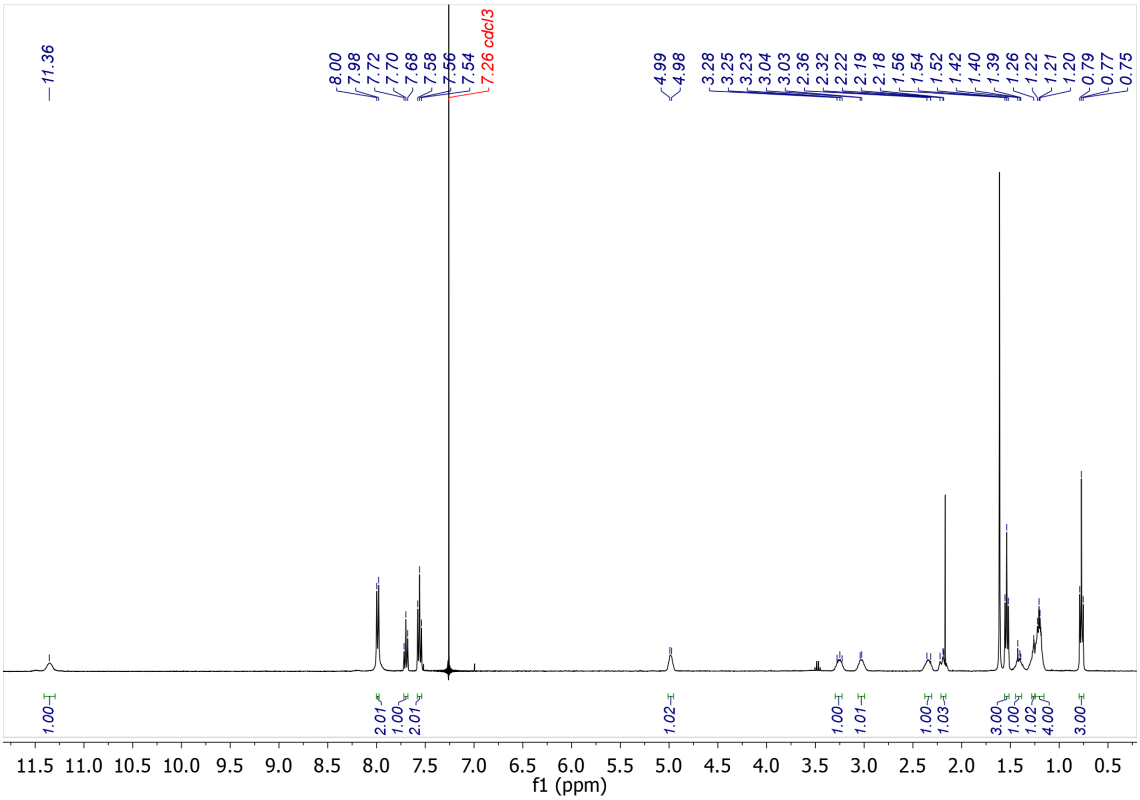


**Figure S17**: $^{1}$H-NMR spectrum of NEHP.

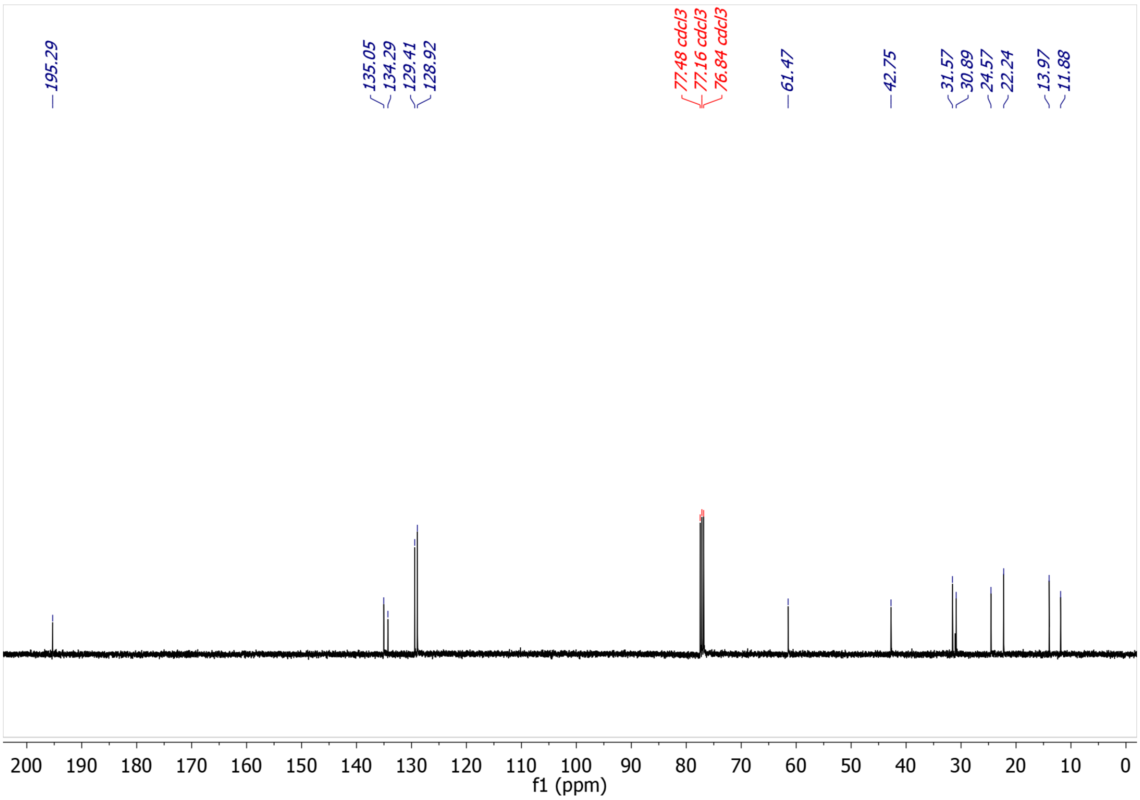


**Figure S18**: $^{13}$C-NMR spectrum of NEHP.

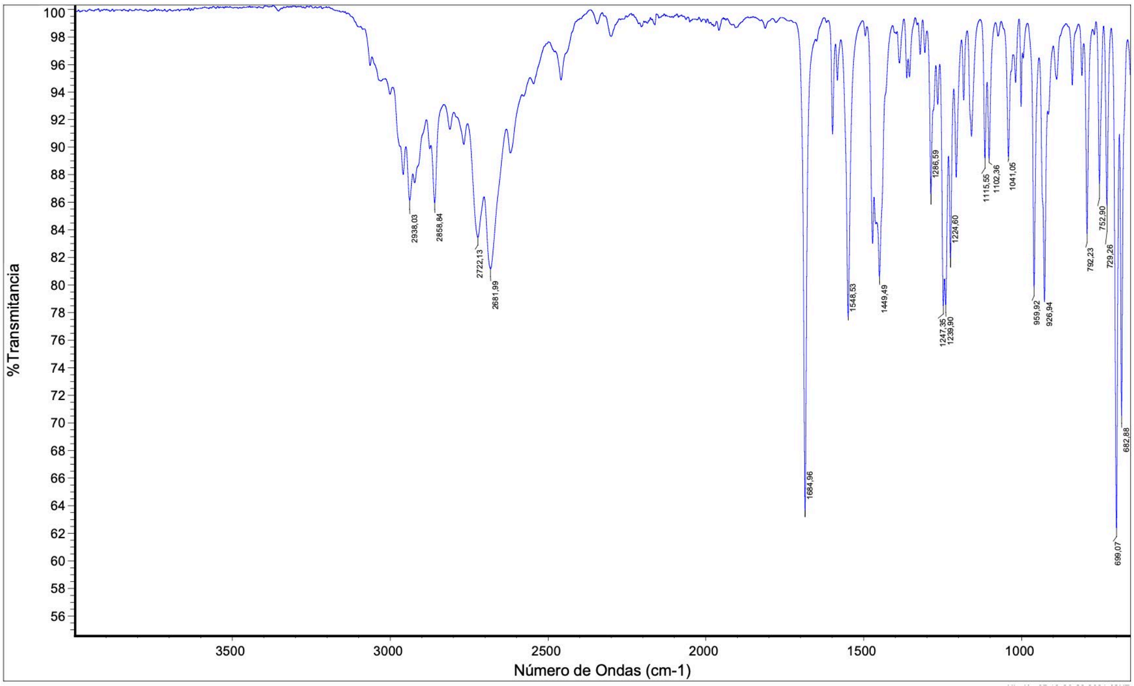


**Figure S19**: IR spectrum of NEHP.

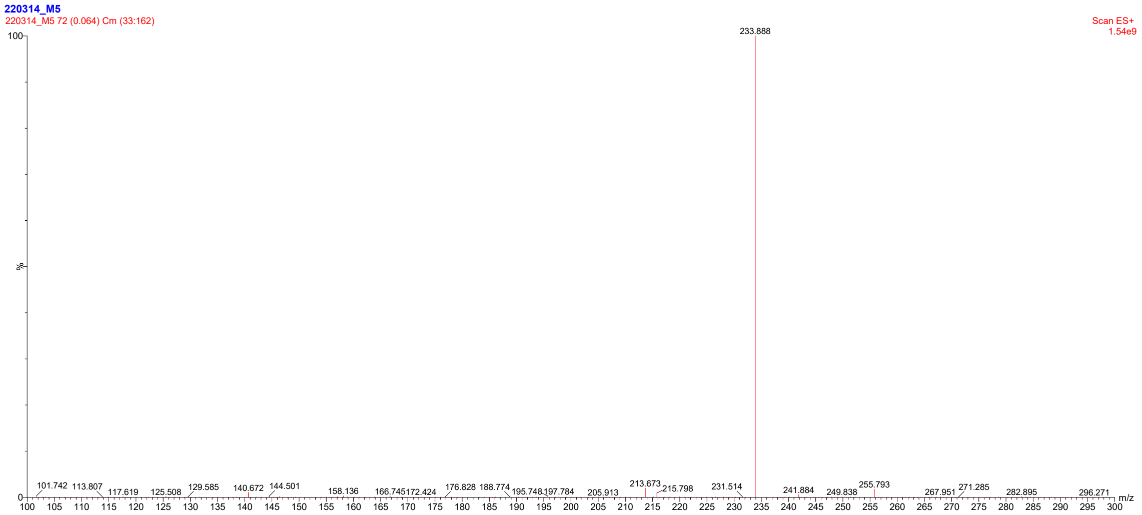


**Figure S20**: MS spectrum of NEHP.

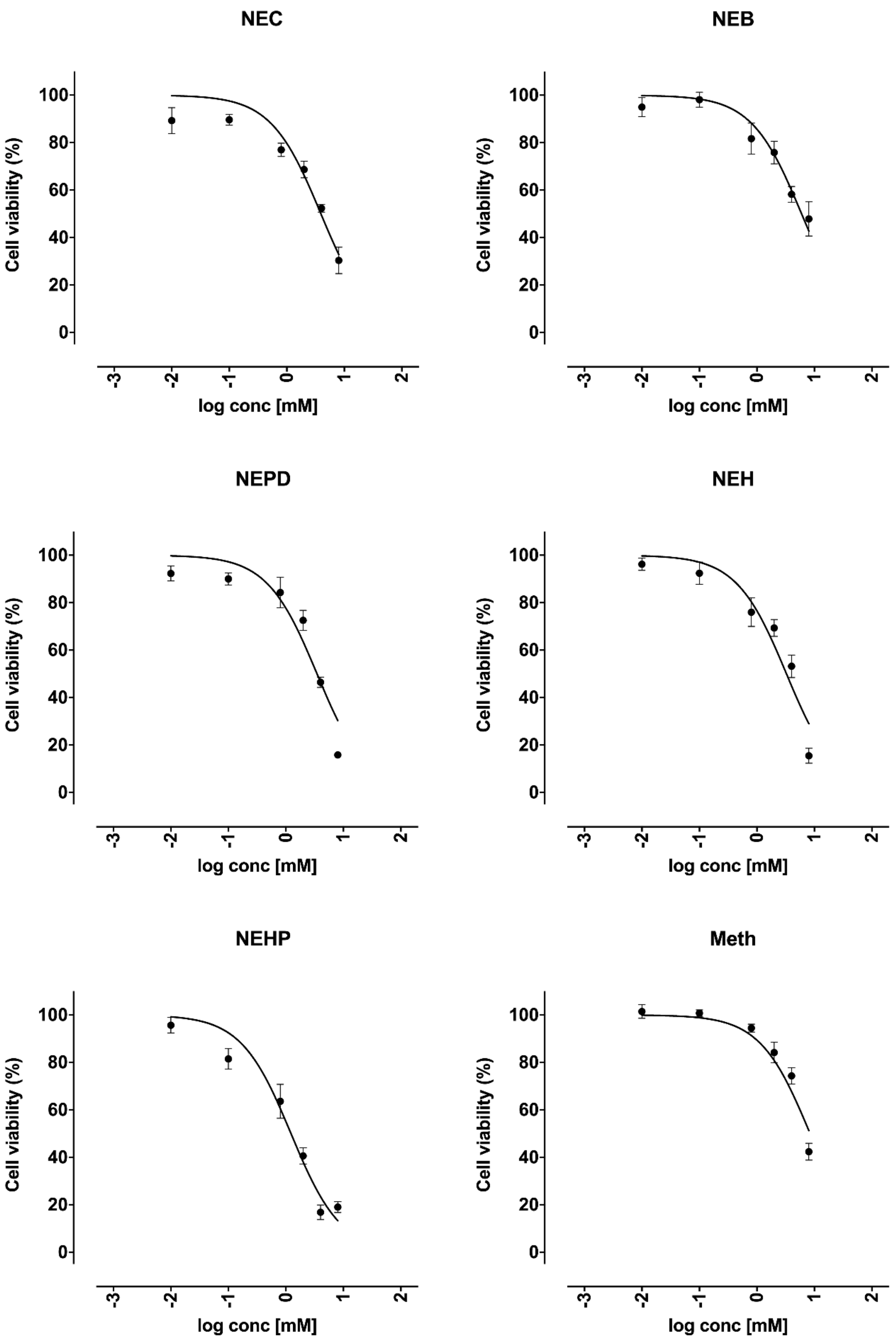


**Figure S21**: Concentration-effect curves on cell viability, expressed in percentage, of the tested cathinones in NGF-differentiated PC12 cells using the WST-8 assay. Data are expressed as a percentage of control cell viability (mean ± SEM) of 3-4 independent experiments carried out on triplicates.
